# Huygens’ clocks revisited

**DOI:** 10.1098/rsos.170777

**Published:** 2017-09-06

**Authors:** Allan R. Willms, Petko M. Kitanov, William F. Langford

**Affiliations:** 1 Department of Mathematics and Statistics, University of Guelph, Guelph Ontario, Canada N1G 2W1; 2 Department of Mathematics and Statistics, University of Ottawa, Ottawa Ontario K1N 6N5

**Keywords:** Huygens' clocks, double Hopf bifurcation, equivariant bifurcation theory, synchronization, nonlinear resonance, coupled identical oscillators

## Abstract

In 1665, Huygens observed that two identical pendulum clocks, weakly coupled through a heavy beam, soon synchronized with the same period and amplitude but with the two pendula swinging in opposite directions. This behaviour is now called anti-phase synchronization. This paper presents an analysis of the behaviour of a large class of coupled identical oscillators, including Huygens' clocks, using methods of equivariant bifurcation theory. The equivariant normal form for such systems is developed and the possible solutions are characterized. The transformation of the physical system parameters to the normal form parameters is given explicitly and applied to the physical values appropriate for Huygens' clocks, and to those of more recent studies. It is shown that Huygens' physical system could only exhibit anti-phase motion, explaining why Huygens observed exclusively this. By contrast, some more recent researchers have observed in-phase or other more complicated motion in their own experimental systems. Here, it is explained which physical characteristics of these systems allow for the existence of these other types of stable solutions. The present analysis not only accounts for these previously observed solutions in a unified framework, but also introduces behaviour not classified by other authors, such as a synchronized toroidal breather and a chaotic toroidal breather.

## 1. Introduction

Christiaan Huygens was a manufacturer of accurate pendulum clocks in the seventeenth century. Huygens was elected a Fellow of The Royal Society in 1663 and he reported there his development of pendulum clocks, with which he sought a solution to the longitude problem posed by The Royal Society [1]. In 1665, in a letter to his father, he reported his observation that two identical clocks hung on a beam synchronized to each other after about 30 min. The motion of the two pendula was such that their periods were identical but their displacements were opposite in direction. Fortunately, this letter has been preserved and is reported in many publications, see [2–8] or [9], appendix A1. At first Huygens thought that this synchronization was caused by air currents, but after further experimentation he concluded that weak coupling of the two clocks through the beam was the cause of this anti-phase synchronization. Today it is recognized that synchronization of oscillators is a fundamental concept in nonlinear science. Recent books that have been devoted to this theme include [9–17]. Many examples of synchronization are given in these references, most of which begin with a review of the synchronization of Huygens' clocks.

Huygens analysed pendulum clocks using sophisticated geometrical arguments in his *Horologium oscillatorium* [18]. After the publication by Isaac Newton of his *Principia* [19], the infinitesimal calculus and differential equations became the preferred mathematical tools for the analysis of such problems, later extended to Lagrangian mechanics in the nineteenth century and eventually to powerful numerical methods in the mid-twentieth century, see [3–8,10,20–28] and further references therein. One of the most complete studies of Huygens' clocks to date is by Bennett *et al.* [3], who used mathematical modelling and numerical computation as well as physical experiments to explore the synchronization of pendulum clocks. Many new studies of Huygens' clocks have appeared since the publication of Bennett *et al.* [3]. Most of these studies report anti-phase synchronization, as observed by Huygens himself. However, some of them report in-phase synchronization; that is, the two clocks move in the same direction at the same time [4,6,7,10,22,24–26]. The analysis presented here explains this discrepancy.

The present paper is a departure from all previous work on Huygens' clocks in that (i) it is the first to fully exploit a symmetry property that is possessed by the system of Huygens' clocks, and (ii) it is the first to apply the methods of equivariant bifurcation theory to this study.

Owing to the great skill of Huygens as a clockmaker, the two clocks in his experiment were essentially identical. Also, the coupling of the two clocks through the beam was symmetrical. Therefore, his experimental system of two clocks and a beam would be unchanged if the two clocks were interchanged. That is, his system possessed a simple permutation symmetry sometimes denoted $Z_{2}$, which we here call Huygens symmetry. It is now known that even such a simple symmetry as this can have a profound effect on the nature of the possible solutions of the system of equations representing the experiment. Previous authors have modelled Huygens' experiment with systems of differential equations that implicitly preserve this symmetry property, but they have not explicitly made use of the symmetry in their analysis. This study is the first to do so (see §3).

This paper employs equivariant bifurcation theory, including the equivariant centre manifold theorem and equivariant normal forms, to classify all of the theoretically possible types of behaviour of a class of systems that includes Huygens' clocks [29–31]. In [31], the normal form for double Hopf bifurcation in a system with Huygens symmetry was derived using Elphick's method. Normal form analysis was performed and different types of solutions were studied in general settings. In this paper, we apply those results to a specific model, that of Huygens' clocks, and analyse the results. In particular, in the present work, a physical model of Huygens' clocks is derived including a model for the escapement mechanism, §2. The importance of symmetry in determining solution types is emphasized in §3 using the Equivariant Hopf Bifurcation Theorem. §4 presents explicit computation of the coefficients and parameters of the normal form in terms of the parameters of the physical model. A detailed description of all of the solution types, a bifurcation analysis, and a stability analysis are presented in §5. The results in this section extend and adapt the results in [31] to the case of Huygens' clocks, augment the stability analysis and clarify the behaviour of the toroidal breather solutions. §6 applies the results to Huygens' experimental situation. Here, it is explained why Huygens saw only anti-phase solutions. In-phase solutions and other behaviour reported by other authors is explained by mapping their physical systems into the same normal form. New types of behaviour and solutions are studied, such as a period-doubling sequence leading from a synchronized breather to a chaotic breather.

## 2. A model of Huygens' clocks

Huygens was an expert clockmaker of his time, and the first to successfully construct a pendulum clock in 1656 [32], pp. 114–118. His invention improved the accuracy of time-keeping devices from the then current 15 min d^−1^, achievable by very good verge-balance wheel clocks, to within 10–15 s d^−1^ [32], p. 116. He was interested in solving the navigational ‘longitude problem’, and his idea was to use his accurate pendulum clock suspended from a rope with a heavy weight in the clock case to keep it upright despite the pitching of the vessel. The clock, set to the appropriate time at the longitude of departure, could then be compared to local time determined from the sun, hence establishing the present longitude of the vessel. To provide some redundancy, in case one clock failed, or when one was being serviced, Huygens realized two identical clocks would be needed; hence the two clocks suspended from a beam in his experiment where he first noted anti-phase synchronization. It turned out his idea was not practically feasible, the rolling of the vessel affecting the pendulum swing despite the heavy weight. It was not until the balance spring was invented in 1675 (in which Huygens also had a part [32], p. 124–126) that an accurate time-keeping device was able to be adapted to maritime use. Our model of Huygens' clock system accounts for the heavy weights in the clock cases by giving the beam a large mass compared to the masses of the pendulum bobs and affixing the clocks rigidly to the beam, as done in Bennett *et al.* [3] and Senator [23]. Senator also studies a 5-degrees of freedom model where the suspension of the clocks from the beam is explicitly modelled, but his analysis suggests this more complicated model provides essentially the same results.

**Figure 1.**
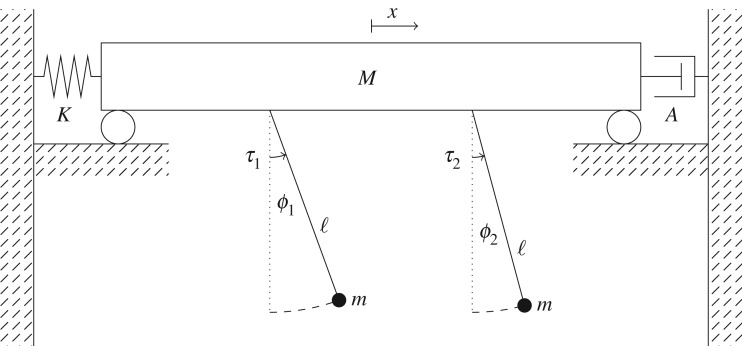
Schematic of the model of Huygens' clocks system.

We model Huygens' clock system as shown in figure 1. Two identical simple pendula, each with mass *m* and length ℓ swing in the same plane and are attached at their pivots to a heavy beam of mass *M*≫*m*. This beam is free to oscillate horizontally in the same plane and is attached to a solid wall via a linear spring with constant *K* and a linear damper with coefficient *A*. The displacement angles of the two pendula are denoted by *ϕ*_*i*_, *i*=1,2, and the displacement of the beam is denoted by *x*. The angles are measured so that a positive angle corresponds to a movement of the pendulum bob in the positive *x*-direction.

The model takes into account both frictional resistance at the pivot and air resistance of the moving bob. Air resistance on the beam itself is incorporated into the linear damping coefficient *A*. Air resistance on each pendulum is assumed to be viscous with coefficient *a*_*a*_, acting in a direction opposite to the velocity of the pendulum bob, which of course depends both on the angular velocity $\dot{\phi_{i}}$ of the swinging pendulum and the horizontal beam velocity, $\dot{x}$. The frictional pivot resistance is proportional to the angular velocity with coefficient *a*_*p*_.

The escapement mechanism provides a torque, $\tau_{i}(\phi_{i},{\dot{\phi }}_{i})$, *i*=1,2, to each pendulum, which is described in the next section.

Under the above assumptions, applying Newton's Laws yields the following set of governing equations for this system, the first two representing the angular momenta of the pendula, and the third the horizontal momentum of the entire system (beam and pendula):

2.1 $$\frac{d}{dt}(m\ell {\dot{\phi }}_{i})=-mg\sin\phi_{i}-m\ddot{x}\cos\phi_{i}-a_{a}\dot{x}\cos\phi_{i}-(a_{a}\ell +a_{p}){\dot{\phi }}_{i}+\frac{\tau_{i}}{\ell },\;i=1,2$$

and

2.2 $$\frac{d}{dt}\left((M+2m)\dot{x}+\sum_{i=1}^{2}m\ell {\dot{\phi }}_{i}\cos\phi_{i}\right)=-A\dot{x}-Kx-2a_{a}\dot{x}+\sum_{i=1}^{2}\left(\frac{\tau_{i}}{\ell }-(a_{a}\ell +a_{p}){\dot{\phi }}_{i}\right)\cos\phi_{i},$$

where *g* is the acceleration due to gravity.

This system has what we call Huygens symmetry: it is unchanged by exchange of the two oscillators. Mathematically, this symmetry is expressed as an invariance under the mapping (*ϕ*_1_,*ϕ*_2_)↦(*ϕ*_2_,*ϕ*_1_), and the same for ${\dot{\phi }}_{i}$. Assuming, as we do below, that the escapement torque, $\tau_{i}(\phi_{i},{\dot{\phi }}_{i})$, is an odd function of its arguments, that is, $\tau_{i}(-\phi_{i},-{\dot{\phi }}_{i})=-\tau_{i}(\phi_{i},{\dot{\phi }}_{i})$, the system possesses an additional symmetry of reflection: (*ϕ*_1_,*ϕ*_2_,*x*)↦(−*ϕ*_1_,−*ϕ*_2_,−*x*). These two symmetries combined we call ‘odd-Huygens symmetry’. Many real pendulum clocks have Huygens symmetry but not odd-Huygens symmetry. The reason is that the escapement mechanism is often not symmetric, pushing the pendulum more in one direction than the other, breaking the odd (reflection) symmetry. The in-phase periodic solutions have Huygens symmetry while the anti-phase periodic oscillations observed by Huygens appear to have what we here call odd-Huygens symmetry. (The system looks the same if we permute the two clocks and simultaneously reverse the displacements and velocities.) Thus, it appears that the pendulum motions have more symmetry than the physical system does. This is referred to as the ‘escapement paradox’. The paradox is resolved by the Equivariant Hopf Bifurcation Theorem (see §3), where it is shown that the anti-phase oscillations have a *spatio-temporal symmetry*, which consists of the above permutation symmetry in space combined with a half-period phase shift in time. This is the true symmetry observed by Huygens.

The above system has a 1 : 1 resonant double Hopf bifurcation point; that is, the linearization at the equilibrium solution, for critical values of the parameters, has complex conjugate pairs of double imaginary eigenvalues (see §3). We assume that there is no further degeneracy, for example in the nonlinear terms. Roughly speaking, an *unfolding* of a bifurcation point is a smooth parametrized family of systems that reduces to the given system at the bifurcation point. An unfolding is called *versal* if every unfolding is (topologically) equivalent to elements of the given unfolding, near the bifurcation point. A versal unfolding is called a *universal unfolding* if it has the minimum number of parameters to be versal. This minimum number of parameters is called the *codimension* of the bifurcation. For most bifurcations of practical interest the codimension is a finite number; for the present case (a system with Huygens symmetry) the codimension is three [31]. However, the model (2.1)–(2.2) of Huygens' clocks is not a versal unfolding, as the following thought experiment shows. In the limit as the mass *M* of the beam tends to infinity, the reaction of the beam to the pendula becomes negligible (the terms in (2.2) involving *ϕ*_*i*_ become insignificant) and the beam will remain stationary. Consequently, the system degenerates in this limit to two identical uncoupled oscillators that undergo simultaneous Hopf bifurcation at the origin when the linear (in ${\dot{\phi }}_{i}$) portion of the forcing term, *τ*_*i*_/ℓ, exceeds the linear damping. For more precise definitions of these terms, see [33], ch. III.

A versal unfolding can be obtained by adding in direct coupling between the pendula. Indeed, Huygens himself first thought that the coupling between his pendula was due to air movement, although he later realized that the significant coupling was occurring through the beam. In any event, by adding direct coupling between the pendula, whether this be due to a damping force (like air movement) or a restoring force (like a spring connecting the two pendulum bobs) the system becomes more general, and versal in the sense of 1–1 resonant Hopf bifurcations. To be versal, we add weak coupling as if both a linear spring with constant *k*≪1 and a linear damper with constant *d*≪1 were present, although just one of these is sufficient to make the system versal. Since the current study is focused on the onset of oscillations from the stationary equilibrium, it is sufficient to approximate the distance between the pendula and the rate of change of this distance as the difference between the pendulum angles and their angular velocities multiplied by the pendulum length, respectively. As in figure 1, we assume that the *x*-coordinate for the pivot of pendulum 2 is more positive than that for the pivot of pendulum 1. Thus, the terms that we add to the right-hand side of (2.1) are

2.3 $${\left(-1\right)}^{i+1}(k\ell (\phi_{2}-\phi_{1})+d\ell ({\dot{\phi }}_{2}-{\dot{\phi }}_{1})),$$

where the ±1 factor is due to the spring and damper acting oppositely on the two pendula. Since this added coupling is symmetric, the system still possesses Huygens symmetry. Our analysis will focus on system (2.1)–(2.2) with the terms (2.3) added; however, when we apply this analysis to the model of Huygens' clocks, the factors *k* and *d* will be set to zero.

### 2.1. Modelling the escapement

**Figure 2.**
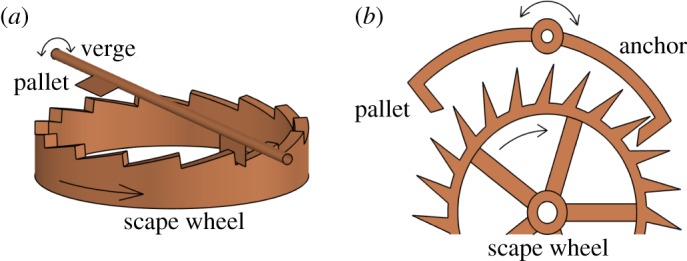
(*a*) Verge escapement mechanism used by the clocks in Huygens' experiments. The scape wheel is mounted horizontally and rotates through connection to an external energy source (not shown). The teeth of the wheel engage with the pallets attached to the verge, which is connected to the pendulum rod. The swinging pendulum thus arrests (and temporarily reverses) the scape wheel movement as each pallet engages a tooth. As the pendulum and verge rotate back, the first pallet disengages and the second pallet engages at the opposite side of the wheel. (*b*) A schematic of an anchor escapement. The scape wheel is mounted vertically in the plane of the pendulum and the anchor pivot is in the same plane above the scape wheel. The anchor pallets engage the teeth but since movement of the pallet is parallel with the tooth face, there is no recoil of the scape wheel. Energy is transferred to the anchor as the tooth slides off the bevelled edge of the pallet face.

Huygens' pendulum clocks worked with a verge escapement mechanism that required pendulum amplitudes of 20° or more [32], p. 119 in order to engage. This introduced circular error, since the period of a pendulum does not remain constant at large amplitude. Huygens showed [34] that suspending the rigid pendulum arm from a cord that was constrained by cycloid-shaped cheeks produced a cycloid arc and hence a period independent of amplitude. However, this invention was superseded by the development of the anchor escapement sometime between 1666 and 1671 [32], p. 120–121, which virtually eliminated circular error by allowing pendulum clocks to function at much smaller amplitudes. In any case though, if the pendulum's amplitude is below that required to engage the escapement mechanism, the clock will not function and the pendulum will come to rest. Figure 2 shows a sketch of both a verge and an anchor escapement mechanism. In both cases, the rotating verge/anchor is attached to a crutch, a long arm ending in a fork that brackets the pendulum rod, thus coupling the swinging pendulum to the rotation of the verge/anchor. See also the diagrams in Landes [32], pp. 362–365.

Our analysis of Huygens' clocks is focused on oscillations arising from the resting state, hence a low-amplitude limit. In order to pursue this analysis, we must approximate the escapement mechanism with one that engages at any amplitude.

All escapement mechanisms transfer energy by means of intermittent contact between the pallets connected by a crutch to the pendulum arm, and the teeth of the escapement wheel. In reality, this transfer of energy is neither purely continuous nor impulsive, but rather a mixture of the two. For both verge and anchor escapements, the tooth of the escapement wheel strikes the pallet but then remains in contact for some time and pushes the pallet. The contact time occupies a significant portion of a period. Researchers have modelled escapement mechanisms using impulsive transfer [3,23] (either at the peak of the arc or at the bottom) piecewise continuous transfer [35,24], or continuous transfer [10,22]. This modelling choice is generally driven by desire for simplicity, whether that be in terms of analysis or numerical computation. Here, we model the contact as continuous so that we can apply the tools of bifurcation theory for systems of ordinary differential equations, and we allow the escapement mechanism to work at all amplitudes. This choice means that our model does not capture the amplitude threshold feature of a true escapement mechanism. In particular, the ‘beating death’ phenomenon reported by Bennett *et al.* [3] and Czolczyński *et al.* [4], which arises when one oscillator transfers so much energy to the other that the first no longer has sufficient amplitude to engage the escapement, is excluded in our model. However, the advantage of our approach is that it allows us to use normal form theory and hence characterize all possible dynamic behaviours of the system near the onset to oscillations.

**Figure 3.**
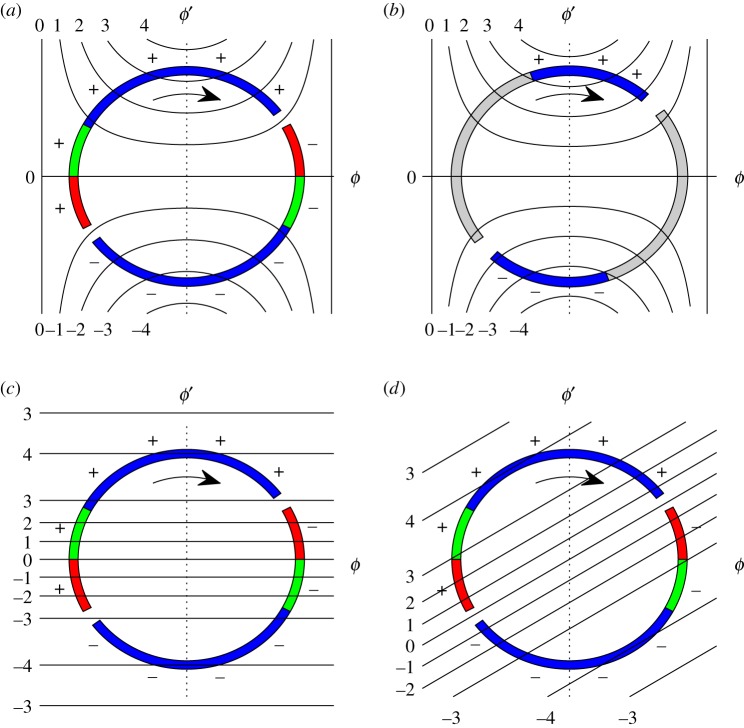
Escapement torque as a function of angular position and (non-dimensionalized) velocity. The pendulum trajectory nearly follows a circle because the friction and forcing are very small. Arrows indicate direction of travel and signs indicate the sign of the torque applied. (*a*) The actual torque for the verge escapement. Recoil, shown in red, occurs when the scape wheel first engages the pallet so that the torque is opposite in sign to *ϕ*′. The green section indicates the region where the pendulum regains the energy lost during the recoil and the blue region is where there is net torque applied to the pendulum. The gaps in the circle are where the pallet disengages with the tooth of the scape wheel and swings free before engaging a tooth on the opposite side of the wheel, providing a torque with opposite sign. Black curves are level sets of the function given in (2.4) used to model the torque. Values (scaled to integers) of the torque level sets are shown on the left, top and bottom. (*b*) Actual torque for the dead beat anchor escapement overlaid with level sets of (2.4). Blue indicates where torque is applied, and grey where the pallet is in contact with the tooth but no torque is applied. (*c*,*d*) Verge escapement overlain with level sets of alternate torque modelling functions, (2.5) and that function rotated.

For a real verge escapement, while a tooth of the wheel is in contact with a pallet, the torque applied to the pallet is a constant. The contact starts prior to the pendulum achieving maximum displacement (zero velocity) and is maintained until well after the pendulum has passed through its equilibrium position (maximum absolute velocity). This is illustrated in figure 3*a*, where we have plotted the trajectory of the pendulum in terms of the displacement, *ϕ*, and the non-dimensional angular velocity, $\phi^{\prime }=\sqrt{(\ell /g})(d\phi /dt)$. Whether the torque is positive or negative when the angular velocity, *ϕ*′, is small depends on whether the pendulum angle is near its negative extreme or its positive extreme. Since the pallet engages prior to the pendulum reaching its maximum displacement, there is a portion of time when the pendulum is working against the escapement wheel driving it backwards (recoil; red portion in figure 3). The energy lost by the pendulum from the point of engagement until maximal displacement (zero velocity) is balanced by the energy gain from the point of maximal displacement until the pendulum has reached the angle (and opposite velocity) at which it was first engaged (green portion in figure 3). Thus, the net energy that is transferred from the escapement mechanism to the pendulum occurs while the pendulum velocity is large in magnitude, with a torque that is constant and of the same sign as the velocity (blue portion in figure 3).

By contrast, a dead beat anchor escapement mechanism, which became the norm by the early 1700s, does not suffer from recoil because the pallet surfaces slide along the scape wheel teeth perpendicularly to the radial direction; see figure 2 and the figure in Landes [32], p. 364. As such, during most of the cycle, there is no torque applied to the pallet by the scape wheel. The applied torque is concentrated near the bottom of the pendulum arc and its amplitude is sensitively dependent on the precise geometry of the scape teeth and curved edges of the pallet surfaces. This situation is illustrated in figure 3*b*.

To model these escapements, we use the function

2.4 $$\tau_{i}=b\phi_{i}^{\prime }-\frac{c}{2}\phi_{i}^{2}\phi_{i}^{\prime },\;i=1,2.$$

Here, *b* and *c* are positive constants chosen so that $2b/c>\phi_{i}^{2}$. The parameter *b* must be large enough for the linear portion of the torque to be greater than the loss due to friction (air resistance and pivot resistance acting on the pendula) because otherwise the pendulum would remain at its rest state. This function for the torque yields Van der Pol oscillators in system (2.1)–(2.2), hence we call it a Van der Pol function. Its choice has two motivations. First, as seen in figure 3*b*, the function given by (2.4) does a good job at continuously approximating the discontinuous anchor escapement torque. Its approximation of the verge escapement torque (figure 3*a*) is not as good but still reasonable. Second, Van der Pol oscillators are well studied and a number of researchers, including Blekhman [10] and Pantaleone [22], have used this form to model the escapement in Huygens' clocks. In an attempt to approximate the verge escapement mechanism more closely, three other torque functions were investigated. The first was a function dependent only on the angular velocity:

2.5 $$\tau_{i}=b\phi_{i}^{\prime }-\frac{c}{6}{\left(\phi_{i}^{\prime }\right)}^{3},\;i=1,2.$$

Here, the parameters *b* and *c* are again positive, and they are chosen so that a significant portion of the pendulum trajectory lies near the local extrema of this function. Again, *b* must be large enough so that the linear portion of the torque is greater than the friction term. Level sets of (2.5) are overlaid on the verge escapement torque in figure 3*c*. The second and third alternate functions had level sets like (2.4) and (2.5), respectively, but rotated in the (*ϕ*,*ϕ*′) plane by about 30°; figure 3*d* illustrates the latter of these two. The rotation of these functions allows us to match the sign of the modelled torque during the recoil phase.

Perhaps surprisingly, it turned out that there was almost no discernible effect on the results regardless of which of these torque functions were used to model the escapement. In addition, the cases with rotation complicated the algebra significantly. For these reasons, we only report the results for the case where the Van der Pol function (2.4) without rotation was used to model the torque. What does make a substantial difference in the model is the relative size of *c* compared to *b*, since this determines the amount of energy input to the pendula and their resulting amplitudes. For a verge escapement the amplitude should be over 20°, while for an anchor escapement a value closer to 8° is more appropriate. For fixed *b*, decreasing the amplitude is achieved by increasing the value of *c*.

### 2.2. Non-dimensional model

The system given by (2.1)–(2.4) may be non-dimensionalized with the transformation

2.6 $$\hat{x}=\frac{x}{\ell }\;\mathrm{and}\;\hat{t}=t\sqrt{\frac{g}{\ell }},$$

where time has been scaled by the undamped natural frequency of the pendula. Applying this change of variables and using the non-dimensional parameters

2.7 $$\begin{array}{lllll} \;\delta =\frac{b/\sqrt{\ell g}-a_{a}\ell -a_{p}}{2m\sqrt{\ell g}}, & \;\epsilon =\frac{m}{M}, & \kappa =\frac{k\ell }{mg}, & & \;\;\eta =\frac{d}{m}\sqrt{\frac{\ell }{g}},\\ \alpha =\frac{a_{a}}{m}\sqrt{\frac{\ell }{g}}, & \beta =\frac{A}{M}\sqrt{\frac{\ell }{g}}, & \gamma =\frac{c}{m\ell g} & \text{and} & \omega^{2}=\frac{K\ell }{Mg}, \end{array}\}$$

system (2.1)–(2.4) becomes

2.8 $$\begin{array}{r} \phi_{i}^{\prime \prime }+{\hat{x}}^{\prime \prime }\cos\phi_{i}=-\sin\phi_{i}-\alpha {\hat{x}}^{\prime }\cos\phi_{i}+2\delta \phi_{i}^{\prime }-\frac{\gamma \phi_{i}^{2}\phi_{i}^{\prime }}{2}+{\left(-1\right)}^{i+1}(\kappa (\phi_{2}-\phi_{1})+\eta (\phi_{2}^{\prime }-\phi_{1}^{\prime })),\;i=1,2 \end{array}$$

and

2.9 $$\begin{array}{r} (1+2\epsilon ){\hat{x}}^{\prime \prime }+\epsilon \sum_{i=1}^{2}\phi_{i}^{\prime \prime }\cos\phi_{i}=-(\beta +2\epsilon \alpha ){\hat{x}}^{\prime }-\omega^{2}\hat{x}+\epsilon \sum_{i=1}^{2}\left[{\left(\phi_{i}^{\prime }\right)}^{2}\sin\phi_{i}+\left(2\delta \phi_{i}^{\prime }-\frac{\gamma \phi_{i}^{2}\phi_{i}^{\prime }}{2}\right)\cos\phi_{i}\right], \end{array}$$

where the prime represents differentiation with respect to $\hat{t}$. The situation in which we are interested is the case where the beam is much more massive than the pendula. We, therefore, consider the limit where *M*, *A* and *K* tend to ∞ at the same rate so that the natural frequency of the beam remains unchanged. Thus, the limit we will investigate is the limit as *ϵ* vanishes while *ω* and *β* remain finite. As shown below, when *ϵ*=0, the origin of the system will undergo a double Hopf bifurcation if (i) the torque cancels the damping effects, that is, when *δ*, which measures the relative size of linear torque to the friction, is zero, and (ii) the direct coupling between the pendula vanishes, that is, *κ* and *η* are zero. Thus *δ*, *ϵ*, *η* and *κ* are the small parameters controllable through the physical constants *M*, *b*, *d* and *k*. The parameter *ω* is the ratio of the natural frequency of the beam to that of the pendula. The parameter *α* is the non-dimensionalized air friction, and *γ* incorporates nonlinear effects of the torque. All eight non-dimensional parameters are non-negative except *δ*, which can take either sign.

The non-dimensional system (2.8)–(2.9) may be considered a linear algebraic system for the three variables *ϕ*_1_′′, *ϕ*_2_′′ and ${\hat{x}}^{\prime \prime }$ in terms of the other quantities. Solving this linear system and then transforming in the usual manner via

2.10 $$y_{1}=\phi_{1},\;y_{2}=\phi_{1}^{\prime },\;y_{3}=\phi_{2},\;y_{4}=\phi_{2}^{\prime },\;y_{5}=\hat{x}\;\mathrm{and}\;y_{6}={\hat{x}}^{\prime },$$

yields the following system of six first-order differential equations:

2.11 $$\begin{array}{rl} y_{1}^{\prime } & \;=y_{2}, \end{array}$$

2.12 y2′ =−sin⁡y1+2δy2−γy12y22 +[ω2y5cos⁡y1+(β−α)y6cos⁡y1+ϵcos⁡y1(sin⁡y1(cos⁡y1+y22)+sin⁡y3(cos⁡y3+y42)) −(1+ϵ(2−cos⁡y3(cos⁡y1+cos⁡y3)))(η(y2−y4)+κ(y1−y3))(sin⁡y1(cos⁡y1+y22)+sin⁡y3(cos⁡y3+y42))][1+2ϵ−ϵ(cos2⁡y1+cos2⁡y3)]−1,

2.13 $$\begin{array}{rl} y_{3}^{\prime } & \;=y_{4}, \end{array}$$

2.14 y4′ =−sin⁡y3+2δy4−γy32y42 +[ω2y5cos⁡y3+(β−α)y6cos⁡y3+ϵcos⁡y3(sin⁡y1(cos⁡y1+y22)+sin⁡y3(cos⁡y3+y42)) −(1+ϵ(2−cos⁡y1(cos⁡y1+cos⁡y3)))(η(y4−y2)+κ(y3−y1))(sin⁡y1(cos⁡y1+y22)+sin⁡y3(cos⁡y3+y42))],[1+2ϵ−ϵ(cos2⁡y1+cos2⁡y3)]−1,

2.15 $$\begin{array}{rl} y_{5}^{\prime } & \;=y_{6} \end{array}$$

2.16 $$\begin{array}{rl} \text{and}\;y_{6}^{\prime } & \;=-\alpha y_{6}+\left[-\omega^{2}y_{5}-(\beta -\alpha )y_{6}+\epsilon (\cos y_{1}-\cos y_{3})(\eta (y_{2}-y_{4})+\kappa (y_{1}-y_{3})\right)\\ & \;\;+\epsilon \left(\sin y_{1}\left(\cos y_{1}+y_{2}^{2}\right)+\sin y_{3}\left(\cos y_{3}+y_{4}^{2}\right)\right)]{\left[1+2\epsilon -\epsilon \left(\cos^{2}y_{1}+\cos^{2}y_{3}\right)\right]}^{-1}. \end{array}$$

The Jacobian of the above system evaluated at the origin when (*δ*,*ϵ*,*η*,*κ*)=(0,0,0,0) is

2.17 $$J=\left[\begin{array}{cccccc} 0 & 1 & 0 & 0 & 0 & 0\\ -1 & 0 & 0 & 0 & \omega^{2} & \beta -\alpha \\ 0 & 0 & 0 & 1 & 0 & 0\\ 0 & 0 & -1 & 0 & \omega^{2} & \beta -\alpha \\ 0 & 0 & 0 & 0 & 0 & 1\\ 0 & 0 & 0 & 0 & -\omega^{2} & -\beta \end{array}\right].$$

The eigenvalues of this matrix are ± *i* (each repeated twice) corresponding to the pendula undergoing simultaneous Hopf bifurcation, and $-\beta /2\pm (i\sqrt{4\omega^{2}-\beta^{2}})/2$, corresponding to the damped oscillations of the beam. The beam is critically damped when *β*=2*ω*, that is, when $A=A_{\mathrm{crit}}:=2\sqrt{MK}$.

## 3. Equivariant Hopf bifurcation

One of the significant mathematical achievements of the latter part of the twentieth century was the development of equivariant bifurcation theory, primarily due to the work of Golubitsky & Stewart [29,30,36,37]. Here, ‘equivariant’ refers to a system with symmetries, for example, Huygens' clocks, as described in the Introduction.

Since no previous investigations of Huygens' clocks have made use of bifurcation theory, we first motivate the relevance of Hopf bifurcation theory for Huygens' clocks. There are many types of bifurcations in mathematics; here, we are concerned only with bifurcations that occur at an equilibrium point of a system of differential equations, such as the six-dimensional (6D) system (2.11)–(2.16) in the previous section. The Jacobian *J* of this system at the equilibrium point y=0 and with (*δ*,*ϵ*,*η*,*κ*)=(0,0,0,0) is given by (2.17). By definition, a bifurcation occurs at y=0 if *J* has either a real eigenvalue λ=0 or a complex conjugate pair of eigenvalues satisfying Re[λ]=0. As det *J*=*ω*^2^>0, it is clear that there is no zero eigenvalue of *J*. However, *J* has double complex conjugate pairs of eigenvalues, {± *i*,± *i*} both with zero real part. This corresponds to a double Hopf bifurcation of the system, provided that further non-degeneracy conditions are satisfied. For double Hopf bifurcation *without* symmetry but with equal eigenvalues, the Jacobian *J* has a *non-semisimple* 4×4 block corresponding to these imaginary eigenvalues as studied in [38]. That is not the case here. In fact, the Huygens symmetry of the system has forced *J* to be semisimple (i.e. diagonalizable over C) as verified in [31]. Thus, the mathematical model of §2 is an example of an equivariant Hopf bifurcation, where the equivariance is given by Huygens permutation symmetry.

There are three defining conditions for equivariant Hopf bifurcation and they have natural physical interpretations in the case of Huygens' clocks. Let us ignore the beam for the moment and consider only the two pendulum clocks. Let $\left\{\lambda_{1},{\bar{\lambda }}_{1},\lambda_{2},{\bar{\lambda }}_{2}\right\}$ be the four eigenvalues corresponding to the Jacobian of the pendulum equations as in (2.11)–(2.14) for small (*δ*,*ϵ*,*η*,*κ*)≠(0,0,0,0). Then, Re[λ_*i*_], *i*=1,2 corresponds to the net linear damping (friction) of each of the pendula for small displacements. One of Huygens' goals in designing these clocks was to keep friction as small as possible; therefore, his system is a small perturbation of

3.1 Re[λi]=0,i=1,2.

Similarly, in order to keep time accurately, Huygens carefully controlled the natural (linear) frequency *Ω* of the two pendula, striving for

3.2 Im[λ1]=Im[λ2]=Ω.

Equations (3.1)–(3.2) are precisely the defining conditions for double Hopf bifurcation. Clearly, the Jacobian in (2.17) satisfies these conditions, with time scaled so that *Ω*=1.

Corresponding to the three defining conditions (3.1)–(3.2) for equivariant double Hopf bifurcation, three independent parameters are necessary to give a *universal unfolding* as defined in the previous section. In other words, equivariant double Hopf bifurcation has *codimension three*. An obvious choice of unfolding parameters for (3.1)–(3.2) is given by {*ϵ*_1_=Re[λ_1_], *ϵ*_2_=Re[λ_2_], *ϵ*_3_=[Im[λ_1_]/Im[λ_2_]−1]}. However, these eigenvalues cannot be controlled directly in experiments. Therefore, one seeks three (or more) experimental parameters that can be controlled directly in the experiments, on which {*ϵ*_1_,*ϵ*_2_,*ϵ*_3_} depend smoothly via functions with rank 3 at (0,0,0). Then, one has a universal (or versal) unfolding of the equivariant double Hopf bifurcation.

Most of the mathematical models in the literature fail this requirement; they do not possess three independent parameters that fully unfold (3.1)–(3.2). That implies that they may be unable to reproduce some of the dynamic behaviour near the bifurcation point. The strength and beauty of bifurcation theory is that it describes the dynamic behaviour of the system, not only at the bifurcation point (defined in this case by (3.1)–(3.2)), but in fact for all possible smooth systems in a full neighbourhood of the bifurcation point. Furthermore, it provides the tools to calculate the solutions, for any particular system such as Huygens' clocks, using the method of centre manifolds and equivariant normal forms. The following §§4–7 of this paper present these calculations for Huygens' clocks.

The Equivariant Hopf Bifurcation Theorem was proved by Golubitsky & Stewart, see [29,30,36,37]. This theorem not only establishes the existence of periodic solutions, it also determines their spatio-temporal symmetries. In order to state this theorem, some definitions are necessary.

In general, consider a real vector space $V=R^{n}$ and assume that *Γ* is a compact Lie group acting on *V* . For *v*∈*V* , the *isotropy subgroup* *Σ*_*v*_⊂*Γ* is the set {*γ*∈*Γ* | *γv*=*v*}. It is easily seen that *Σ*_*v*_ is a subgroup of *Γ*. Similarly, for any subgroup *Σ*⊂*Γ*, define the *fixed point subspace* of *Σ* as Fix(*Σ*)={*v*∈*V* | *σv*=*v* ∀ *σ*∈*Σ*}. Again, it is easy to show that Fix(*Σ*) is a subspace of *V* .

Now, let *F*(*v*,*μ*) be a smooth function, $F\;:\;R^{n}\times R\to R^{n}$, satisfying *F*(0,*μ*)=0, and with non-singular Jacobian *J*=(∂*F*/∂*v*)(0,0). We assume that *F* is *Γ*-*equivariant*, that is

3.3 F(γv,μ)=γF(v,μ),∀ γ∈Γ.

On linearizing equation (3.3) at (0,0), we find

3.4 Jγ=γJ,∀ γ∈Γ.

We assume, for Hopf bifurcation, that *J* has purely imaginary eigenvalues ±i*ω*_0_, which have multiplicity *m* and corresponding critical eigenspace *E* of dimension 2*m*≤*n*. As *J* commutes with *Γ* by (3.4), the critical eigenspace *E* is *Γ*-invariant. Furthermore, *E* is invariant for the solutions (flow) of the differential equations and the original nonlinear system has an invariant centre manifold that is tangent to *E* at the origin. Let *J*|_*E*_ denote the restriction of *J* to the eigenspace *E* and define an *S*^1^-action on *E* by

3.5 $$\theta \to \exp\left(\frac{\theta }{\omega_{0}}J{|}_{E}\right),\;\theta \in [0,2\pi ).$$

Then, *E* is also *Γ*×*S*^1^-invariant. We assume further that *E* is *Γ*-*simple*; this is a technical condition that is satisfied generically, see [29, ch. XVI, proposition 1.4].

Now, consider the Jacobian *J*(*μ*)≡(∂*F*/∂*v*)(0,*μ*) for *μ*≈0. Under the present assumptions, for small *μ*, *J*(*μ*) has eigenvalues *σ*(*μ*)±*iω*(*μ*) each of multiplicity *m* and depending smoothly on *μ*, with *σ*(0)=0, *ω*(0)=*ω*_0_; for proof see [29, ch. XVI, lemma 1.5]. Finally, let *Σ*⊂*Γ*×*S*^1^ be an isotropy subgroup of the action of *Γ*×*S*^1^ restricted to the critical eigenspace *E*.

**Theorem 3.1 (Equivariant Hopf Bifurcation Theorem)**

*Under the above conditions, if*

3.6 dim Fix(Σ)=2

*and*

3.7 $$\sigma^{\prime }(0)\neq 0,$$

*then there exists a branch of periodic solutions parametrized by μ, bifurcating from (0,0), with spatio-temporal symmetry group Σ⊂Γ×S*^1^*. On the periodic solutions, S*^1^ *acts by phase shifts* θ:t→t+θ(T/2π)*, where T is the period, and T≈2π/ω*_0_.

For the proof of this theorem, see [29], ch. XVI, theorem 4.1. Note that, after restriction to the two-dimensional fixed point subspace (3.6), this becomes essentially the classical Hopf bifurcation theorem (codimension one). The ‘crossing condition’ (3.7) is satisfied generically; the case where (3.7) fails to hold is investigated thoroughly in [39].

Unfortunately, this theorem does not apply directly to the model equations presented here for Huygens' clocks (2.11)–(2.16). The problem is that the *Γ*-*simple* hypothesis is not satisfied. However, for the symmetry group of Huygens' clocks, the proof of the theorem is easily modified and the same conclusions hold, even without the *Γ*-*simple* hypothesis (M Golubitsky & I Stewart 2017, personal communication).

Now apply this (modified) theorem to the model of Huygens' clocks, where *m*=2 from (2.17). The spatial symmetry group has two elements, *Γ*={1,*χ*}, where *χ* is permutation of the two clocks. There are two non-trivial subgroups of *Γ*×*S*^1^, each of order 2, namely

$$\Sigma_{1}=\{(1,0),(\chi ,0)\}\;\mathrm{and}\;\Sigma_{2}=\{(1,0),(\chi ,\pi )\}.$$

Both subgroups have fixed point subspaces of dimension 2 as in (3.6) and the crossing condition (3.7) is satisfied. Therefore, there are two branches of periodic solutions; in-phase periodic solutions corresponding to *Σ*_1_ and anti-phase periodic solutions corresponding to *Σ*_2_. Classically, these in-phase and anti-phase solutions are called *normal modes*. Although both exist in the model equations, one can expect to observe them in experiments only if they are *stable* (more precisely, if they are asymptotically orbitally stable). The stabilities of these solutions are calculated in §5.3 and then compared with other theoretical and experimental results in the literature.

The authors previously calculated these in-phase and anti-phase solutions directly in [31], using centre manifold reduction and normal forms. Therefore, the existence of these solutions is not in doubt.

The significance of Equivariant Bifurcation Theory for Huygens' clocks is that it answers the fundamental question that was asked by Huygens, 350 years ago: *Why* did he observe anti-phase synchronization of his two identical clocks? Generations of investigators have attempted to answer this question, using mechanical models, differential equation models and numerical simulations. To the knowledge of the present authors, none of these investigators considered the role of symmetry in Huygens' system. Most of these investigators were able to reproduce Huygens' observations, but none explained *why* he observed what he did, that is, why such anti-phase solutions exist to be observed in the first place. The fundamental answer is now revealed. The anti-phase solutions exist because of the particular spatio-temporal symmetry possessed by Huygens' system. Equivariant bifurcation theory tells us this, without any need to solve the equations or to construct mechanical models. Golubitsky and Stewart were the first to recognize the importance of spatio-temporal symmetries in problems like this one.

## 4. Centre manifold and normal form calculations

The following computations were performed on system (2.11)–(2.16). First, a linear change of coordinates was applied to the system to put the Jacobian in real Jordan form, and then the centre manifold, tangent to the (*y*_1_,*y*_2_,*y*_3_,*y*_4_,*δ*,*ϵ*,*η*,*κ*)-hyperplane was computed by the method outlined in [40] to order 3. The reduced system on this centre manifold was computed to order 3 in the variables *y* and order 1 in (*δ*,*ϵ*,*η*,*κ*). Huygens symmetry is manifest in the resulting four-dimensional (4D) reduced system by the vector field being equivariant with respect to the mapping

4.1 $$x\mapsto \left[\begin{array}{cc} 0 & I_{2}\\ I_{2} & 0 \end{array}\right]x,$$

where *I*_2_ is the 2×2 identity matrix. The 4D reduced system was then subject to a linear transformation dependent on (*δ*,*ϵ*,*η*,*κ*) so that the linear part of the new system remains diagonal as these parameters are varied. Finally, a near-identity transformation with cubic terms was applied to remove the unnecessary cubic terms from the vector field yielding the normal form as given in [41,31]. The result, after keeping only first-order terms in (*δ*,*ϵ*,*η*,*κ*), is the topologically equivalent complex system defined by

4.2 $$\begin{array}{rl} {\dot{z}}_{1} & \;=(\delta -C_{1}\epsilon +i(1-C_{2}\epsilon ))z_{1}+\frac{1}{4}(-\gamma +2[2C_{1}+C_{3}+3\gamma C_{4}]\epsilon \\ & \;\;+i(-1+3\gamma \delta +[-\gamma C_{1}+9C_{2}-2\gamma C_{3}+6C_{4}]\epsilon ))z_{1}^{2}z_{3}\\ & \;\;+\frac{1}{4}(-\gamma +2[2C_{1}-\gamma C_{2}-C_{3}+\gamma C_{4}]\epsilon -2\gamma \kappa \\ & \;\;+i(-1+3\gamma \delta +[-3\gamma C_{1}+5C_{2}+2\gamma C_{3}+2C_{4}]\epsilon -2\gamma \eta ))z_{2}^{2}z_{3}\\ & \;\;+\frac{1}{2}(-\gamma +2[2C_{1}+C_{3}+\gamma C_{4}]\epsilon \\ & \;\;+i(-1+3\gamma \delta +[\gamma C_{1}+5C_{2}-2\gamma C_{3}+2C_{4}]\epsilon -2\gamma \eta ))z_{1}z_{2}z_{4} \end{array}$$

and

4.3 $$\begin{array}{rl} {\dot{z}}_{2} & \;=(\delta -\eta +i(1+\kappa ))z_{2}+\frac{1}{4}(-\gamma +2[\gamma C_{2}+2C_{3}+2\gamma C_{4}]\epsilon +2\gamma \kappa \\ & \;\;+i(-1+3\gamma \delta +2[\gamma C_{1}+2C_{2}-2\gamma C_{3}+2C_{4}]\epsilon -\gamma \eta +\kappa ))z_{1}^{2}z_{4}\\ & \;\;+\frac{1}{4}(-\gamma +i(-1+3\gamma \delta -3\gamma \eta +\kappa ))z_{2}^{2}z_{4}\\ & \;\;+\frac{1}{2}(-\gamma +4\gamma C_{4}\epsilon +i(-1+3\gamma \delta +2[-\gamma C_{1}+2C_{2}+2C_{4}]\epsilon -\gamma \eta +\kappa ))z_{1}z_{2}z_{3}, \end{array}$$

where $[z_{1},z_{2},z_{3},z_{4}]=[z_{1},z_{2},\bar{z_{1}},\bar{z_{2}}]\in C^{4}$, and the parameters *C*_*j*_ are given in terms of the non-dimensional parameters by

4.4 $$\begin{array}{rlrl} C_{0} & \;={\left(\omega^{2}-1\right)}^{2}+\beta^{2}, & C_{1} & \;=\frac{\alpha (\omega^{2}-1)+\beta }{C_{0}},\;C_{2}=\frac{\omega^{2}-1-\alpha \beta }{C_{0}},\\ C_{3} & \;=\frac{\omega^{2}(\omega^{2}-1)C_{1}+\beta (C_{2}+1)}{C_{0}}\;\text{and} & C_{4} & \;=\frac{(\omega^{4}-1)C_{2}-1}{C_{0}}. \end{array}\;\}$$

The coordinate *z*_1_ corresponds to in-phase motion, where the angles and velocities of both pendula are identical, while *z*_2_ corresponds to anti-phase motion, where the angle and velocity of the second pendulum are negative that of the first. The quantity *C*_0_, appearing in the denominators of the other parameters *C*_*j*_, is a measure of distance from resonance. If *ω*≈1 (the beam and pendula have nearly the same natural frequency) and *β* is small (the beam damping is small), then *C*_0_ is close to zero and, consequently, the parameters *C*_*j*_, *j*>1, will be large. In this case, for the first-order Taylor approximations in (4.2)–(4.3) to be valid *ϵ* (the pendulum to beam mass ratio) would need to be very small. The analysis below will exclude this near-resonance region, so that *ω* is sufficiently different from one and/or the beam damping is sufficiently large.

Define polar coordinates, *z*_1_=*r*_1_ e^i*θ*_1_^ and *z*_2_=*r*_2_ e^i*θ*_2_^. As shown in [41,31], the vector field for the real 4D polar coordinate system depends only on the difference of the polar angles, not on the angles themselves. Therefore, following [41,31], define the quantities

4.5 $$\begin{array}{rlrl} \psi_{1} & \;=\arg(\mathrm{Coef}(z_{2}^{2}z_{3})), & \psi_{2} & \;=\arg(\bar{\mathrm{Coef}(z_{1}^{2}z_{4})}),\\ \theta & \;=2(\theta_{1}-\theta_{2})-\psi_{1}\;\text{and} & \psi & \;=\psi_{1}-\psi_{2}, \end{array}\}$$

where arg⁡(Z) is the argument of the complex number *Z*, and Coef(*U*) is the coefficient of *U* in system (4.2)–(4.3). Rewriting the complex system (4.2)–(4.3) in terms of these amplitude-phase normal mode coordinates results in a 4D real system that has cubic terms $a_{11}r_{1}^{3}$ and $a_{22}r_{2}^{3}$ in the ${\dot{r}}_{1}$ and ${\dot{r}}_{2}$ equations, respectively, where

4.6 $$a_{11}=-\frac{\gamma }{4}+\frac{1}{2}(2C_{1}+C_{3}+3\gamma C_{4})\epsilon \;\text{and}\;a_{22}=-\frac{\gamma }{4}.$$

Assuming that *a*_11_<0, then scaling the amplitudes via

4.7 $${\hat{r}}_{1}=\sqrt{-a_{11}}r_{1}\;\text{and}\;{\hat{r}}_{2}=\sqrt{-a_{22}}r_{2},$$

results in (after dropping the hats) the following 4D normal form system:

4.8 $$\begin{array}{rl} {\dot{r}}_{1} & \;=r_{1}[\mu_{1}-r_{1}^{2}+(b_{12}+B_{1}\cos(\theta ))r_{2}^{2}], \end{array}$$

4.9 $$\begin{array}{rl} {\dot{r}}_{2} & \;=r_{2}[\mu_{2}+(b_{21}+B_{2}\cos(\theta +\psi ))r_{1}^{2}-r_{2}^{2}], \end{array}$$

4.10 $$\begin{array}{rl} \dot{\theta_{1}} & \;=c_{10}+c_{11}r_{1}^{2}+(c_{12}-B_{1}\sin(\theta ))r_{2}^{2} \end{array}$$

4.11 $$\begin{array}{rl} \text{and}\;\dot{\theta_{2}} & \;=c_{20}+(c_{21}+B_{2}\sin(\theta +\psi ))r_{1}^{2}+c_{22}r_{2}^{2}. \end{array}$$

The normal form for the corresponding three-dimensional (3D) real system (in terms of *θ* rather than *θ*_1_ and *θ*_2_) is

4.12 $$\begin{array}{rl} {\dot{r}}_{1} & \;=r_{1}[\mu_{1}-r_{1}^{2}+(b_{12}+B_{1}\cos(\theta ))r_{2}^{2}], \end{array}$$

4.13 $$\begin{array}{rl} {\dot{r}}_{2} & \;=r_{2}[\mu_{2}+(b_{21}+B_{2}\cos(\theta +\psi ))r_{1}^{2}-r_{2}^{2}] \end{array}$$

4.14 $$\begin{array}{rl} \text{and}\;\dot{\theta } & \;=2[\mu_{3}+(b_{31}-B_{2}\sin(\theta +\psi ))r_{1}^{2}+(b_{32}-B_{1}\sin(\theta ))r_{2}^{2}], \end{array}$$

where

4.15 $$\begin{array}{rl} c_{10} & \;=1-C_{2}\epsilon ,\;\;c_{20}=1+\kappa , \end{array}$$

4.16 $$\begin{array}{rl} c_{11} & \;=-\frac{1}{\gamma }+3\delta +\frac{1}{\gamma^{2}}(-(4+\gamma^{2})C_{1}+9\gamma C_{2}-2(1+\gamma^{2})C_{3})\epsilon , \end{array}$$

4.17 $$\begin{array}{rl} c_{12} & \;=-\frac{2}{\gamma }+6\delta +\frac{2}{\gamma }(\gamma C_{1}+5C_{2}-2\gamma C_{3}+2C_{4})\epsilon -4\eta , \end{array}$$

4.18 $$\begin{array}{rl} c_{21} & \;=-\frac{2}{\gamma }+6\delta +\frac{4}{\gamma^{2}}(-(2+\gamma^{2})C_{1}+2\gamma C_{2}-C_{3}-\gamma C_{4})\epsilon -2\eta +\frac{2\kappa }{\gamma }, \end{array}$$

4.19 $$\begin{array}{rl} c_{22} & \;=-\frac{1}{\gamma }+3\delta +-3\eta +\frac{\kappa }{\gamma }, \end{array}$$

4.20 $$\begin{array}{rl} \mu_{1} & \;=\delta -C_{1}\epsilon ,\;\;\mu_{2}=\delta -\eta ,\;\;\mu_{3}=c_{10}-c_{20}=-C_{2}\epsilon -\kappa , \end{array}$$

4.21 $$\begin{array}{rl} b_{12} & \;=-2+\frac{4}{\gamma }(2C_{1}+C_{3}+\gamma C_{4})\epsilon ,\;\;b_{21}=-2-\frac{4}{\gamma }(2C_{1}+C_{3}+\gamma C_{4})\epsilon , \end{array}$$

4.22 $$\begin{array}{rl} B_{1} & \;=(1+\gamma^{2}-3\gamma \delta +[-\gamma C_{1}+(-5+2\gamma^{2})C_{2}-2(1+\gamma^{2})C_{4}]\epsilon \\ & \;\;+2\gamma \eta +2\gamma^{2}\kappa ){(\gamma \sqrt{1+\gamma^{2}})}^{-1}, \end{array}$$

4.23 B2 =(1+γ2−3γδ+2γ[(2+γ2)C1−γ(2+γ2)C2+(1+γ2)C3+γ(1+γ2)C4]ϵ2γ+γη−(1+2γ2)κ)(γ1+γ2)−1,

4.24 $$\begin{array}{rl} b_{31} & \;=c_{11}-c_{21}\\ & \;=\frac{1}{\gamma }-3\delta +\frac{1}{\gamma^{2}}((4+3\gamma^{2})C_{1}+\gamma C_{2}+2(1-\gamma^{2})C_{3}+4\gamma C_{4})\epsilon +2\eta -\frac{2\kappa }{\gamma } \end{array}$$

4.25 $$\begin{array}{rl} \text{and}\;b_{32} & \;=c_{12}-c_{22}\\ & \;=-\frac{1}{\gamma }+3\delta +\frac{2}{\gamma }(\gamma C_{1}+5C_{2}-2\gamma C_{3}+2C_{4})\epsilon -\eta -\frac{\kappa }{\gamma }. \end{array}$$

A necessary condition for the above to be valid is the requirement that *a*_11_ given by (4.6) is negative, so that the scaling given by (4.7) is well defined. If this condition fails, then the negative sign on the $r_{1}^{3}$ term in (4.12) must be changed to a positive sign. Since the constants *C*_*i*_, 1≤*i*≤4, all have a *C*_0_ in the denominator (4.4), this condition fails when *ϵ*/*C*_0_ is too big compared with *γ*/4. This will only occur when the system is both near resonance, *ω*≈1, and the beam damping, *β*, is small, but that case is excluded herein. As *a*_22_ given by (4.6) is clearly always negative, there is no additional condition to impose.

With the conditions *a*_11_<0 and *a*_22_<0 satisfied, yielding system (4.12)–(4.14), both primary bifurcations (the in-phase and anti-phase normal modes) are supercritical and stable. There are three other cases. The case in which both primary bifurcations are subcritical (*a*_11_ and *a*_22_ both positive) can be transformed into this case by changing the signs of both *t* and *μ*. The remaining two cases, in which one normal mode bifurcation is supercritical and the other is subcritical, lead to more complex behaviour and are not considered here.

The power of the normal form (4.12)–(4.14) is that *all* models with a 1–1 resonant double Hopf bifurcation in the presence of Huygens symmetry and with *a*_11_*a*_22_>0 have the same dynamics near this bifurcation as exhibited by the normal form. This is not to say that any such model will necessarily display all the dynamics found in the normal form, but that the normal form captures all possible dynamical behaviour of any such model. The specific dynamics of any particular model depend on the mapping from the physical parameters to the parameters in the normal form. For the case of the model given by (2.8)–(2.9), this mapping is given by (4.4)–(4.5), (4.15)–(4.25).

The normal form (4.12)–(4.14) has been truncated to cubic terms and the effect of higher-order terms on the following analysis has not been rigorously established. It is our expectation that all of the solution types and bifurcations described in §5 persist with the addition of higher-order terms except perhaps the period doubling cascade discussed there. All of the other solutions are simple equilibria or periodic orbits of the 3D system and are expected to be structurally stable, hence will be qualitatively unaffected by higher-order terms.

The parameters *μ*_1_, *μ*_2_ and *μ*_3_ are the unfolding parameters for the bifurcation depending on the four small non-dimensional parameters *δ*, *ϵ*, *η* and *κ*. If there is no direct coupling between the pendula (*η*=*κ*=0), then variation of *δ* and *ϵ* would only move the system away from the bifurcation in a 2D manifold in the 3D *μ*-space. This is the manifestation of the degeneracy of the system mentioned above, which is removed when direct coupling between the pendula is introduced. For sufficiently small values of *δ*, *ϵ*, *η* and *κ*, the parameters *b*_12_, *b*_21_ and *b*_32_ will be negative, while *B*_1_, *B*_2_ and *b*_31_ will be positive. Indeed, if one ignores the *δ*, *ϵ*, *η* and *κ* contributions to the parameters of the higher-order terms, the system simplifies to

4.26 $$\begin{array}{rl} {\dot{r}}_{1} & \;=r_{1}\left[\mu_{1}-r_{1}^{2}+\left(-2+\frac{\sqrt{1+\gamma^{2}}}{\gamma }\cos(\theta )\right)r_{2}^{2}\right], \end{array}$$

4.27 $$\begin{array}{rl} {\dot{r}}_{2} & \;=r_{2}\left[\mu_{2}+\left(-2+\frac{\sqrt{1+\gamma^{2}}}{\gamma }\cos(\theta +\psi )\right)r_{1}^{2}-r_{2}^{2}\right] \end{array}$$

4.28 $$\begin{array}{rl} \text{and}\;\dot{\theta } & \;=2\left[\mu_{3}+\left(\frac{1}{\gamma }-\frac{\sqrt{1+\gamma^{2}}}{\gamma }\sin(\theta +\psi )\right)r_{1}^{2}+\left(-\frac{1}{\gamma }-\frac{\sqrt{1+\gamma^{2}}}{\gamma }\sin(\theta )\right)r_{2}^{2}\right]. \end{array}$$

## 5. Basic solutions and bifurcations of the normal form

In this section, we present a summary of the solution types and basic bifurcations of the 3D normal form system (4.12)–(4.14), or equivalently (4.26)–(4.28). The results of [31] are extended and applied to the model of Huygens' clocks. The stability analysis in §5.3 is new and the behaviour of the toroidal breather solutions is clarified. The analysis of this 3D normal form system demonstrates the existence of a variety of solutions, including trivial and non-trivial equilibrium points, periodic orbits and aperiodic solutions. Each of these solutions of the 3D system (4.12)–(4.14) corresponds to a (typically more complicated) solution of the 4D system (4.8)–(4.11) in amplitude-phase normal mode coordinates. Ultimately, this leads to a solution of the 6D model system (2.11)–(2.16) in physical coordinates. Thus, the 3D system (4.12)–(4.14) is fundamental to understanding the dynamic behaviour of our model of Huygens' clocks and is the basis for the results in the following sections of this paper.

### 5.1. Basic solutions

It is important to note that the state space of the 3D system (4.12)–(4.14) (or (4.26)–(4.28)) is

5.1 $$(r_{1},r_{2},\theta )\in R^{+}\times R^{+}\times S^{1},$$

where *R*^+^ denotes the non-negative real numbers and *S*^1^ is the real line modulo 2*π*. Let us begin with the obvious *trivial solution* (*r*_1_,*r*_2_)=(0,0) with *θ* arbitrary, in the 3D system (4.12)–(4.14) above. Clearly, this corresponds to trivial solutions of both the 4D and 6D systems; both normal modes and both pendula have zero amplitude of oscillation. The trivial solution is stable only if *μ*_1_ and *μ*_2_ are both negative. Suppose that the parameter *μ*_1_ increases through zero. Then, a new solution bifurcates from the trivial solution, given by $\left(r_{1},r_{2})=(\sqrt{\mu_{1}},0\right)$, on the positive *r*_1_ axis. This is the *in-phase normal mode periodic solution*, for which the two pendula swing in unison, with the same amplitude and phase. Similarly, $\left(r_{1},r_{2})=(0,\sqrt{\mu_{2}}\right)$ bifurcates from the trivial solution on the positive *r*_2_ axis as *μ*_2_ increases through 0, and corresponds to the *anti-phase normal mode periodic solution*. These primary normal mode bifurcations are pitchfork bifurcations of the 3D system (4.12)–(4.14) (actually semi-pitchfork bifurcations because *r*_1_ and *r*_2_ are non-negative amplitudes), but they become Hopf bifurcations of periodic solutions from the trivial solution in the 4D and 6D systems, as was predicted in §3 using the Equivariant Hopf Bifurcation Theorem.

For both of the normal mode solutions, the value of *θ* is irrelevant so far as the 4D system is concerned, because *θ* is the difference between the two polar angles *θ*_*i*_ and one of the polar amplitudes *r*_*i*_ is zero, meaning the corresponding *θ*_*i*_ is undefined. Yet, *θ* is well defined as a solution of the 3D system. For each of the normal modes, whether $\left(r_{1},r_{2},\theta )=(\sqrt{\mu_{1}},0,\theta \right)$, or $\left(0,\sqrt{\mu_{2}},\theta \right)$, the $\dot{\theta }$-equation (4.14) (or (4.28)) has two distinct types of solutions in the state space (5.1), as described in the following two cases:

*Phantom Case* 1. There exists a pair of equilibria (*r*_1_,*r*_2_,*θ*_*s*/*u*_), with *θ*_*s*_≠*θ*_*u*_, satisfying $\dot{\theta }=0$. The coordinates (*r*_1_,*r*_2_) are either $\left(\sqrt{\mu_{1}},0)\;\mathrm{or}\;(0,\sqrt{\mu_{2}}\right)$, as in the previous paragraph. The point with *θ*_*s*_ is stable and with *θ*_*u*_ is unstable, in the *θ*-direction. These two equilibria are connected by heteroclinic orbits that are constant in the coordinates (*r*_1_,*r*_2_) and vary only in *θ*.
*Phantom Case* 2. There exists a periodic orbit satisfying $\dot{\theta }\neq 0$. Then, $\dot{\theta }$ is always of one sign and *θ*(*t*) loops through *S*^1^ modulo 2*π*. Coordinates (*r*_1_,*r*_2_) remain constant as above, either $\left(\sqrt{\mu_{1}},0\right)$ or $\left(0,\sqrt{\mu_{2}}\right)$.

Although these two cases correspond to qualitatively different solutions of the 3D system (4.12)–(4.14), they are indistinguishable for solutions of the 4D system (4.8)–(4.11). The transitions between Case 1 and Case 2 are saddle-node bifurcations on a *θ*-cycle and correspond to crossing the boundaries of an Arnold tongue for the $\dot{\theta }$ equation as shown in [31]. Since these bifurcations are not present in the 4D or 6D models, we call them ‘phantom bifurcations’. Nonetheless, they have significant effects on other solutions and secondary bifurcations of the system.

In Case 1 above ($\dot{\theta }=0$), in which a normal mode is represented as a pair of equilibrium points on a boundary *r*_*i*_=0 of the state space (5.1), the normal mode may undergo a secondary pitchfork bifurcation, giving rise to an equilibrium point in the interior of (5.1). At this new equilibrium point both normal mode amplitudes satisfy *r*_*i*_>0, so it represents a mixture of the two modes. The condition $\dot{\theta }=0$ is preserved locally, implying that the phase difference of the two normal modes, *θ*=2(*θ*_1_−*θ*_2_)−*ψ*_1_, is a constant. In the corresponding 4D system, this equilibrium point is a periodic solution where both normal mode amplitudes and the phase difference are constant. In the physical 6D system, this solution corresponds to the pendula oscillating at the same frequency but at different phases, and with constant but different amplitudes. Therefore, we call these *mixed-mode periodic solutions*. As shown in [41,31], in the interior of the state space (5.1), the 3D system (4.12)–(4.14) may have up to four equilibrium points, with both *r*_*i*_>0, *i*=1,2, and $\dot{\theta }=0$, giving up to four mixed-mode periodic solutions.

**Figure 4.**
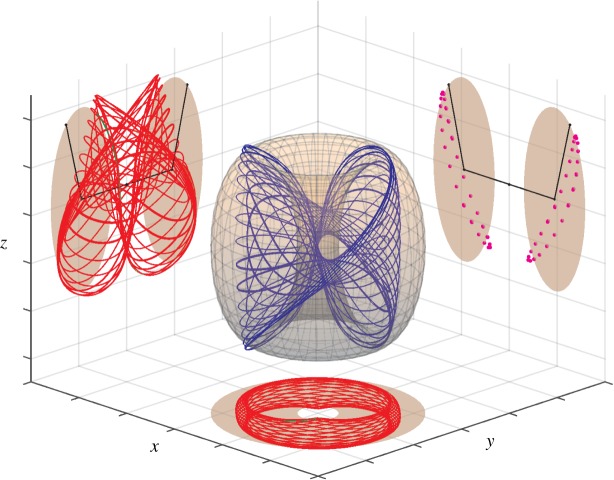
Example of a synchronized toroidal breather solution. The blue central trajectory is a projection into 3-space via $x=(r_{1}+r_{2}\cos\theta_{2})\cos\theta_{1}$, $y=(r_{1}+r_{2}\cos\theta_{2})\sin\theta_{1}$, $z=r_{2}\sin\theta_{2}$, of the stable solution of (4.8)–(4.11) with parameter values given by (6.4) and (6.5), except that *μ*_1_=0.06, *μ*_2_=0.1675, *μ*_3_=0.175, *c*_10_=1.175, *c*_20_=1 and *ψ*_1_=0. Two planar projections (red) of this trajectory are shown on the left and bottom coordinate planes. The Poincaré map for *y*=0 (magenta) is shown on the right coordinate plane. Note that the Poincare map appears to be restricted to a one-dimensional closed curve. The breathing torus and its cross sections at *x*=0, *y*=0 and *z*=0 (brown) are shown as they appear for the final point of the trajectory. This figure is the last frame of the ‘synchronized breather’ video in [42].

In Case 2 above ($\dot{\theta }\neq 0$), a normal mode solution (with one of *r*_1_=0 or *r*_2_=0) again may undergo a bifurcation that results in both *r*_*i*_>0. Because in this case both the normal mode solution and the 3D system (4.12)–(4.14) are 2*π*-periodic in *θ*, this bifurcation is not a simple steady-state pitchfork as in Case 1, but rather a pitchfork bifurcation for a periodic orbit (or equivalently for the Poincaré map), which can be analysed using Floquet theory or averaging, see below. The new solution is again a periodic solution of the 3D system that continually wraps around in *θ* (modulo 2*π*), but now exists in the interior of the state space (5.1). In the corresponding 4D system, both *θ*_*i*_ are defined, and as $\dot{\theta }\equiv 2({\dot{\theta }}_{1}-{\dot{\theta }}_{2})$ is of one sign, one of *θ*_1_ or *θ*_2_ continually laps the other modulo 2*π*. As the frequencies of *θ*_1_ and *θ*_2_ are not rationally related, in general, the result in the 4D system is a quasi-periodic orbit. In the physical system, this solution is characterized by the pendula exchanging energy between one another so that their amplitudes vary in a quasi-periodic fashion. We call these solutions *toroidal breathers* for the following reason. As shown in [31] and illustrated by videos in [42] and a similar video in [43], these solutions of the 4D system may be projected into 3-space and represented as trajectories on a 2-torus where the two amplitudes of the torus, *r*_*i*_, both vary slowly in *θ* with period 2*π*. The visual result is one of a trajectory riding on the surface of a ‘breathing’ 2-torus; for example, in figure 4. Although it is not easy to show the ‘breathing’ behaviour of the torus in a two-dimensional (2D) figure, it becomes quite clear in the time-lapse video in [42]. In figure 4, the trajectory appears to be restricted to a 2D manifold (the 2-torus) that self-intersects. These self-intersections are not real, but are a consequence of the projection from the 4D system.

There are two distinct types of behaviour of the solutions on this toroidal breather. For the first type of behaviour, the solutions (*r*_1_,*r*_2_,*θ*_1_,*θ*_2_) are 2*π*-periodic in *θ*. The solutions are synchronized with the 2*π*-periodic parametric forcing. In this case, as the 2-torus ‘breathes’ with period 2*π* in *θ*, the solutions do the same, and are restricted to a 2D manifold in state space, a distorted 2-torus. A Poincaré section reveals that the Poincaré map is restricted to a closed curve. This is the case illustrated in figure 4 and the first video in [42], as well as in [31]. We call this case a *synchronized toroidal breather*. The existence of these solutions was proved in [41,31] using Brouwer degree and a theorem of Krasnoselskii. The proof need not be repeated here, but briefly, it uses the fact that $\dot{\theta }\neq 0$ implies that ±*θ* is a time-like variable, and the 3D normal form system (4.12)–(4.14) can be reduced to a 2D system with *θ* as the independent variable. However, the 2D system is now non-autonomous, with 2*π*-periodic parametric forcing; see equations (5.21)–(5.22) in §5.3. Then, theorem 5.1.10 in [41] or theorem 5.3 in [31] states that there exist open regions in parameter space and in state space in which these equations have a solution that is 2*π*-periodic in *θ*. From this fact follows easily the existence of the synchronized breather, as seen in figure 4.

**Figure 5.**
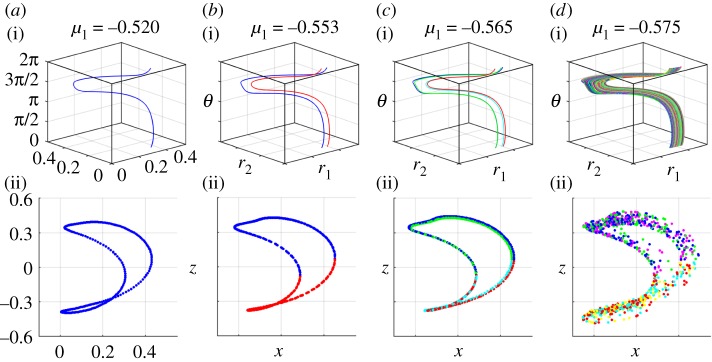
Period doubling sequence leading from a synchronized breather to a chaotic breather. (*a*(i)–*d*(i)) Solutions of system (4.12)–(4.14) and the parameter values given by (6.6), *μ*_2_=0.416916, *μ*_3_=0.710259 and values of *μ*_1_ as specified above each plot. (*a*(i)) The synchronized breather as a periodic solution in the 3D system (*r*_1_,*r*_2_,*θ*) with *μ*_1_=−0.520. The orbit leaves the top surface, *θ*=2*π*, and re-enters at the bottom, *θ*=0. (*b*(i)) *μ*_1_=−0.553, the orbit has undergone a period doubling bifurcation. (*c*(i) and *d*(i)) By *μ*_1_=−0.565, a second such bifurcation has occurred and by *μ*_1_=−0.575 many more such bifurcations have occurred and the orbit is apparently chaotic. (*a*(ii)–*d*(ii)) Poincaré maps of the cross section *θ*_1_=0 of the 4D system (4.8)–(4.11) in projected coordinates (*x*,*z*) with parameter values given by (6.6) and *c*_11_=*c*_22_=*b*_32_, *c*_12_=*c*_21_=2*b*_32_. The first period doubling does not result in a doubling of the curve on the map because, due to the definition of *θ*, on the cross section *θ*_1_=0, *θ*_2_ can only differ by modulo *π* as *θ* increases through 2*π*. Colours of points in these plots correspond to the trajectory portions shown in (*a*(i)–*d*(i)).

When the cited existence theorem fails to hold, the solutions (*r*_1_,*r*_2_,*θ*_1_,*θ*_2_) are no longer required to be synchronized with the parametric forcing as above, i.e. no longer 2*π*-periodic in *θ*. In this case, the solutions could escape the 2D invariant manifold seen in figure 4, which would thicken in a third dimension. Similarly, the cross section for the Poincaré map would thicken from a closed curve to a 2D annulus. One example of this case is shown in figure 5. Here, the periodic orbit in the 3D system representing the synchronized breather undergoes a period doubling cascade, resulting in an apparently chaotic orbit. Inspection of the numerical computation of the orbit of the Poincaré map suggests that it may be the product of a Cantor set with a line, an indication of chaotic dynamics. We call this case a *chaotic toroidal breather*. A video of a chaotic breather can be found in [42].

It is well known that, for the generic Hopf–Hopf bifurcation, in the case where *a*_11_*a*_22_<0, the 2-torus with both *r*_1_>0 and *r*_2_>0 may undergo a tertiary Hopf bifurcation, leading to a 3-torus with a small third frequency [40,44,45]. In the present case of equivariant Hopf bifurcation, the truncated normal form (4.8)–(4.11) reduces (up to order 3) to that of generic Hopf–Hopf bifurcation in the limit as *B*_1_ and *B*_2_ vanish. To see the 3-torus in the generic case, fifth-order terms are necessary. The fifth-order terms in the present case will be different from those in the generic case; nonetheless, one may expect to find similar behaviour in this case if *B*_1_ and *B*_2_ are small enough. However, the persistence of such a 3-torus is non-generic [46–48] and may lead to more complicated dynamics on a ‘strange attractor’. These issues are not explored further here because, from (4.6), *a*_11_*a*_22_<0 is not possible in the case of Huygens' clocks, but such behaviour may be important for other cases of equivariant Hopf bifurcation.

The last primary solution type arises when an equilibrium point in the interior of the state space (5.1) (corresponding to a mixed-mode periodic solution) undergoes a Hopf bifurcation. This gives rise to a periodic orbit of the 3D system around the equilibrium point, where the *θ* values remain bounded (do not lap modulo 2*π*). In the 4D system, this Hopf bifurcation becomes a torus bifurcation, and the corresponding solution trajectory winds around the original mixed-mode periodic orbit on the surface of a thin 2-torus. Again, since the frequencies of *θ*_1_ and *θ*_2_ are not in general rationally related, this motion on the 2-torus is quasi-periodic. For the physical system, the amplitudes and the difference in the angular displacements of the two pendula vary quasi-periodically around the values corresponding to the original mixed-mode periodic solution. We call these solutions *mixed-mode 2-torus solutions*. The primary difference in the 3D system between these solutions and the breather solutions is that the normal mode phase difference, *θ*, remains bounded for these solutions, that is, does not lap modulo 2*π*. The manifest difference in the physical system is that the pendula amplitudes and phase difference vary more substantially for the breather solutions than for these solutions (at least near where these solutions first appear).

More exotic solutions than these also exist for (4.12)–(4.14), some of which are described below. However, the types of solutions listed above are the principal stable solutions over most of the parameter space.

### 5.2. Secondary bifurcations

The analysis of the secondary bifurcations leading to the above-described solutions begins as follows. Consider the in-phase normal mode solution of the 3D system (4.12)–(4.14), that is $\left(r_{1},r_{2},\theta )=(\sqrt{\mu_{1}},0,\theta \right)$. (The analysis for the anti-phase mode is analogous.) Assume first Case 1 of the ‘phantom bifurcations’ above, at the stable solution with *θ*=*θ*_*s*_. Then, the Jacobian matrix for the 3D system at $\left(r_{1},r_{2},\theta )=(\sqrt{\mu_{1}},0,\theta_{s}\right)$ is

5.2 $$J=\left[\begin{array}{ccc} -2\mu_{1} & 0 & 0\\ 0 & \mu_{2}+[b_{21}+B_{2}\cos(\theta_{s}+\psi )]\mu_{1} & 0\\ 4\sqrt{\mu_{1}}(b_{31}-B_{2}\sin(\theta_{s}+\psi )) & 0 & -2B_{2}\mu_{1}\cos(\theta_{s}+\psi ) \end{array}\right].$$

Let us denote the diagonal elements of *J* by {λ_1_,λ_2_,λ_3_}, respectively. As *J* is triangular, these are the eigenvalues of *J*. A zero eigenvalue of *J* corresponds to a secondary bifurcation. Note that λ_1_≡−2*μ*_1_<0 on the in-phase normal mode branch, implying asymptotic stability and no bifurcation in the *r*_1_ direction. A secondary bifurcation that gives rise to a solution with *r*_2_>0 will occur when

5.3 $$\lambda_{2}(\theta_{s})\equiv [\mu_{2}+b_{21}\mu_{1}]+B_{2}\cos(\theta_{s}+\psi )\mu_{1}=0.$$

Because *r*_2_ is an amplitude coordinate, the secondary bifurcation corresponding to condition (5.3) is one-half of a pitchfork bifurcation, and because *a*_22_<0, (4.6), so that the coefficient of the $r_{2}^{3}$ term in (4.13) is −1, the new solution (with *r*_2_>0) bifurcates supercritically in *μ*_2_.

Finally,

5.4 $$\lambda_{3}(\theta_{s})\equiv -2B_{2}\cos(\theta_{s}+\psi )\mu_{1}=0$$

corresponds to the ‘phantom’ saddle-node bifurcations described above, where two equilibrium points of the $\dot{\theta }$ equation,

5.5 $$\frac{\dot{\theta }}{2}=[\mu_{3}+b_{31}\mu_{1}]-B_{2}\sin(\theta_{s/u}+\psi )\mu_{1}=0,$$

coalesce and vanish on the boundary of an Arnold tongue. Note that (5.5) determines the value of *θ*_*s*_ in (5.3) or (5.4). Eliminate *θ*_*s*_ from (5.3) using (5.5) by squaring and adding to get

5.6 $${\left(\mu_{2}+b_{21}\mu_{1}\right)}^{2}+{\left(\mu_{3}+b_{31}\mu_{1}\right)}^{2}={\left(B_{2}\mu_{1}\right)}^{2}.$$

This represents a cone in the (*μ*_1_,*μ*_2_,*μ*_3_)-parameter space, with radius *B*_2_*μ*_1_>0 and centre on the line (*μ*_1_,*μ*_2_,*μ*_3_)=*μ*_1_(1,−*b*_21_,−*b*_31_) for each *μ*_1_>0. We call this the ‘in-phase cone’. The significance of this cone is that a secondary bifurcation from the in-phase normal mode to a mixed-mode phase-locked solution occurs on crossing this cone. (This crossing of the cone must avoid neighbourhoods of the two lines where the cone intersects the plane (5.8) below. These are lines of codimension-two bifurcations, as shown below.) Similar analysis leads to an ‘anti-phase cone’ for bifurcations from the anti-phase normal mode.

In the same manner, simultaneously solving equations (5.4) and (5.5) and eliminating *θ*_*s*_ by squaring and adding gives two linear equations

5.7 $$\mu_{3}+b_{31}\mu_{1}=\pm B_{2}\mu_{1},\;\mu_{1}>0,$$

which determine where the phantom saddle-node bifurcations occur. These are the boundaries of the Arnold tongue, represented in (*μ*_1_,*μ*_2_,*μ*_3_) space as two planes, tangent to the corresponding cone on the line *μ*_2_+*b*_21_*μ*_1_=0 [31]. The cone exists completely between these two planes.

Outside of this tongue, and therefore outside of the in-phase cone, we are in Case 2 above, with $\dot{\theta }\neq 0$ in (5.5). (In fact, $\dot{\theta }>0$ for *μ*_3_ above the upper plane in (5.7) and $\dot{\theta }<0$ below the lower plane.) For the 3D system, the in-phase normal mode is 2*π* periodic in *θ*, but this is a ‘phantom period’ for the 4D system. Even so, we may investigate the possibility of secondary bifurcations from this periodic solution of the 3D system. A standard tool for the study of bifurcations from a periodic solution is *Floquet theory*. Briefly, if a real Floquet exponent changes sign, then for the present system there is a pitchfork bifurcation from the original periodic orbit, creating a pair of periodic orbits of which only the one with *r*_2_>0 is relevant. The calculation of this real Floquet exponent was presented in [31], §6. Alternatively, the method of averaging may be used as in §5.3 below. The result of either calculation is that a bifurcation of a mixed-mode periodic orbit from the in-phase normal mode in Case 2 occurs on crossing the plane

5.8 $$\mu_{2}+b_{21}\mu_{1}=0,\;\mu_{1}>0,$$

outside of the cone. We call this set the ‘in-phase plane’. (The condition *μ*_1_>0 is required for existence of the in-phase normal mode.) On crossing this plane, a pitchfork bifurcation for orbits gives birth to the toroidal breather described above in Case 2. The in-phase plane meets the cone at the two vertical extremes (in *μ*_3_) given by (5.7). The portion of the in-phase plane that is inside the in-phase cone is not relevant, and so, when we say ‘in-phase plane’, we shall mean only the portion that lies outside the in-phase cone.

The lines of intersection of the in-phase plane and in-phase cone are also where the phantom bifurcation planes meet the cone; the meeting of the phantom bifurcation planes and the cone is tangential. These intersection lines are lines of codimension-two double zero bifurcations; both λ_2_(*θ*_*s*_), (5.3), and λ_3_(*θ*_*s*_), (5.4), are zero. As the system is invariant on changing the sign of *r*_2_, this double zero has flip symmetry. Its normal form is listed in Chow *et al.* [49], p. 178, who give bifurcation diagrams and phase portraits for four different cases. It turns out for this system that only their cases (I) and (III) apply, [49], p. 337ff.. In case (I), between the phantom bifurcation planes the equilibria $\left(\sqrt{\mu_{1}},0,\theta_{s/u}\right)$ exist, and outside the in-phase cone the mixed-mode equilibrium exists. In the narrow regions between these tangentially meeting surfaces all three equilibria exist. Other than the bifurcations giving rise to these equilibria at the phantom bifurcation planes and the in-phase cone, there are no other nearby bifurcations. In case (III), outside both the phantom bifurcation planes and the in-phase cone no equilibria exist, while inside these surfaces all three equilibria exist. In addition, emanating from the intersection of these surfaces and extending inside the cone, there is a surface of heteroclinic connections where a trajectory connects the equilibria $\left(\sqrt{\mu_{1}},0,\theta_{s/u}\right)$, and there is a surface of Hopf bifurcations of the mixed-mode equilibrium.

Similar statements can be made about anti-phase normal mode bifurcations at the anti-phase cone:

5.9 $${\left(\mu_{1}+b_{12}\mu_{2}\right)}^{2}+{\left(\mu_{3}+b_{32}\mu_{2}\right)}^{2}={\left(B_{1}\mu_{2}\right)}^{2},\;\mu_{2}>0$$

and the anti-phase plane:

5.10 $$\mu_{1}+b_{12}\mu_{2}=0,\;\mu_{2}>0,$$

outside of the anti-phase cone. The parameters *b*_12_ and *b*_21_ dictate the orientation of the in-phase and anti-phase planes, respectively.

The mixed-mode periodic solutions that are born at the surface of the in-phase and anti-phase cones may annihilate each other in a secondary saddle-node bifurcation. The mixed-mode periodic solutions are defined by setting the expressions in square brackets on the right-hand sides of equations (4.12)–(4.14) to zero. The first two of these equations are linear in $r_{1}^{2}$ and $r_{2}^{2}$ and hence can be easily solved for these two quantities. Substituting these resulting expressions for $r_{1}^{2}$ and $r_{2}^{2}$ into the right-hand side of (4.14) yields an equation of the form *h*(*θ*)=0. A saddle-node bifurcation generically exists when both *h*(*θ*)=0 and *h*′(*θ*)=0. These two equations can be solved to yield a parametric representation of the surface of saddle-node bifurcations in *μ*-space:

5.11 $$\mu_{1}=\mu_{3}\frac{N_{2}(\theta )D^{\prime }(\theta )-N_{2}^{\prime }(\theta )D(\theta )}{N_{1}(\theta )N_{2}^{\prime }(\theta )-N_{1}^{\prime }(\theta )N_{2}(\theta )}$$

and

5.12 $$\mu_{2}=\mu_{3}\frac{-N_{1}(\theta )D^{\prime }(\theta )+N_{1}^{\prime }(\theta )D(\theta )}{N_{1}(\theta )N_{2}^{\prime }(\theta )-N_{1}^{\prime }(\theta )N_{2}(\theta )},$$

where

$$\begin{array}{rl} D(\theta ) & \;=1-(b_{12}+B_{1}\cos(\theta ))(b_{21}+B_{2}\cos(\theta +\psi )),\\ N_{1}(\theta ) & \;=b_{31}-B_{2}\sin(\theta +\psi )+(b_{32}-B_{1}\sin(\theta ))(b_{21}+B_{2}\cos(\theta +\psi )),\\ N_{2}(\theta ) & \;=b_{32}-B_{1}\sin(\theta )+(b_{31}-B_{2}\sin(\theta +\psi ))(b_{12}+B_{1}\cos(\theta )). \end{array}$$

These surfaces are tangent to, and terminate at the in-phase and anti-phase cones. Expressions for the values of *θ* at which this tangential intersection occurs are given in §5.3 below, and in [31]. Typically, these saddle-node surfaces delimit a region between the cones where mixed-mode periodic solutions exist.

**Figure 6.**
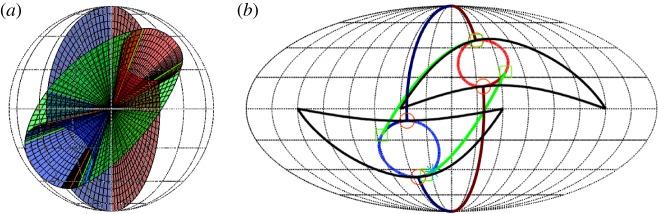
Bifurcation diagram for system (4.12)–(4.14) in *μ*-space showing bifurcations of the normal modes and creation/destruction of mixed-mode solutions. (*a*) 3D plot (the view is from the positive *μ*_1_=*μ*_2_ direction and *μ*_3_ is the vertical) showing the in-phase, on the left (blue), and anti-phase, on the right (red), cones; the in-phase and anti-phase vertical planes that bisect these cones; and the two surfaces of saddle-node bifurcations of mixed-mode equilibria that join these cones together (green). (*b*) Mollweide projection of a sphere centred at the origin in parameter space. The central meridian is the plane *μ*_1_=*μ*_2_>0. The in-phase and anti-phase cones appear roughly as circles in the lower left and upper right of the diagram, respectively. The in-phase and anti-phase planes, because they have constant longitude, are arcs of ellipses emanating from the north and south poles and terminating at the respective cones in a double zero bifurcation point marked with a (red) circle. The curves joining the two cones and tangent to them are the surfaces of saddle-node bifurcations of mixed-mode equilibria. The tangency, marked with a (green) square, is a degenerate pitchfork bifurcation for (4.12)–(4.14), marking transitions of the pitchfork bifurcations on the cones from supercritical to subcritical. This super/subcriticality also changes at the double zero bifurcations. The (light blue) star marks a Bogdanov-Takens point on the curve of saddle-node bifurcations of the mixed-mode equilibria. The phantom bifurcations of the normal mode solutions are shown in this plot (but not in (*a*)) as crescent-shaped curves (black) surrounding the two cones.

All of the above bifurcation varieties may be graphed in (*μ*_1_,*μ*_2_,*μ*_3_) parameter space, as shown in figure 6. The features illustrated in figure 6*a* are the two cones (in-phase and anti-phase), the two corresponding vertical planes bisecting these cones and two surfaces of saddle-node bifurcations of mixed-mode equilibria that connect the cones. The surfaces of ‘phantom’ saddle-node bifurcations in *θ* are not shown in figure 6*a*, but are illustrated in the projection shown in figure 6*b*. The surfaces of codimension-one bifurcations can intersect in curves of codimension-two bifurcations, including those of Bogdanov–Takens type and of Bautin type.

Figure 6 displays the situation where both *b*_12_ and *b*_21_ are negative and their product is less than one. These parameters together with *b*_31_ and *b*_32_ also determine the centre lines of the in-phase and anti-phase cones. The parameters *B*_2_ and *B*_1_ control the radii of the in-phase and anti-phase cones, respectively. In figure 6, all of these values have been chosen to yield a good visualization of the cones and planes. In §6, the bifurcation diagram for parameter values chosen to correspond to Huygens' clocks is presented. In that case, the planes switch sides (the product *b*_12_*b*_21_ is greater than one) and the cones are very fat (*B*_1_ and *B*_2_ are large), and overlap each other a small amount.

Figure 6*a* is further simplified in figure 6*b*. As system (4.12)–(4.14) is invariant under the scaling

$$\mu \mapsto \frac{\mu }{∥\mu ∥},\;r_{1}\mapsto \frac{r_{1}}{\sqrt{∥\mu ∥}},\;r_{2}\mapsto \frac{r_{2}}{\sqrt{∥\mu ∥}}\;\mathrm{and}\;t\mapsto t∥\mu ∥,$$

it follows that the bifurcation diagram is the same on every sphere centred at the origin in *μ*-space. Figure 6*b* shows the Mollweide projection [50] of a sphere centred at the origin, corresponding to the 3D bifurcation diagram in figure 6*a*. This projection, often used for the earth, is an equal area projection where the centre circle is the front hemisphere and the back hemisphere is split vertically and represented by the two wings to the left and right of the central circle. Lines of latitude appear as horizontal lines, and lines of longitude as half of an ellipse joining the north and south poles.

### 5.3. Stability analysis

Readers interested in only the application of the normal form to the phenomena of Huygens' clocks may skip this section.

In [31], the authors analysed the normal form (4.12)–(4.14). The analysis there focused primarily on the case *b*_12_*b*_21_<1, with only some comments on the case in question here, namely *b*_12_*b*_21_>1. We will expand on that analysis in this section by looking at the stability of bifurcating solutions that come from crossing the in- and anti-phase planes and cones for system (4.12)–(4.14). This section places no restriction on the parameters of the system other than that *B*_1_ and *B*_2_ must be positive [31]. In particular, it does not assume the Huygens' clocks values of the parameters given by (4.20)–(4.25). The reader is referred to [31] for the necessary background.

#### 5.3.1. Bifurcations at the cone

First, consider the case where *μ*_2_ increases through the in-phase cone (5.6) passing through a point (*μ*_10_,*μ*_20_,*μ*_30_) on the cone. At this point, one of the equilibria representing the in-phase normal mode, namely either $\left(r_{1},r_{2},\theta )=(\sqrt{\mu_{10}},0,\theta_{s}\right)$ or $\left(r_{1},r_{2},\theta )=(\sqrt{\mu_{10}},0,\theta_{u}\right)$, undergoes a pitchfork bifurcation. It is not difficult to show that the equilibrium with *θ*=*θ*_*s*_, that is, the equilibrium that is fully stable, bifurcates on the half of the cone that has *μ*_2_ values below the in-phase plane, (5.8), and that $\cos(\theta_{s}+\psi )>0$ on this half of the cone. Likewise, the equilibrium with *θ*=*θ*_*u*_, which is stable except for in the *θ*-direction, bifurcates on the other half of the cone, with *μ*_2_ values above the in-phase plane, and on this half of the cone, $\cos(\theta_{u}+\psi )<0$.

The pitchfork bifurcation gives birth to two new equilibria, one with *r*_2_<0, hence unphysical, and one mixed-mode periodic solution with *r*_2_>0. As discussed in [31], the sub- or supercriticality of this bifurcation depends both on which side of the in-phase plane one is, that is, whether the equilibrium with *θ*=*θ*_*s*_ or *θ*=*θ*_*u*_ is bifurcating, and on the location of the intersection with the cone of a surface of saddle-node bifurcations of two mixed-mode equilibria. Here, we show explicitly the type of bifurcation occurring and justify the comments in [31]. Allow *μ*_2_ to vary slightly away from *μ*_20_ and look for equilibrium solutions of (4.12)–(4.14) as Taylor series in (*μ*_2_−*μ*_20_) centred at the in-phase normal mode $\left(r_{1},r_{2},\theta )=(\sqrt{\mu_{10}},0,\theta_{s/u}\right)$:

5.13 $$\begin{array}{rl} r_{1}^{2} & \;=\mu_{10}+A_{1}(\mu_{2}-\mu_{20})+\cdots , \end{array}$$

5.14 $$\begin{array}{rl} r_{2}^{2} & \;=A_{2}(\mu_{2}-\mu_{20})+\cdots \end{array}$$

5.15 $$\begin{array}{rl} \text{and}\;\theta & \;=\theta_{s/u}+A_{3}(\mu_{2}-\mu_{20})+\cdots . \end{array}$$

The result for the first-order coefficients is

5.16 $$\begin{array}{rl} A_{1} & \;=\frac{\left(b_{12}+B_{1}\cos(\theta_{s/u}))\cos(\theta_{s/u}+\psi \right)}{D_{I}}, \end{array}$$

5.17 $$\begin{array}{rl} A_{2} & \;=\frac{\cos(\theta_{s/u}+\psi )}{D_{I}} \end{array}$$

5.18 $$\begin{array}{rl} \text{and}\;A_{3} & \;=\frac{\left(b_{12}+B_{1}\cos(\theta_{s/u}))(b_{31}-B_{2}\sin(\theta_{s/u}+\psi ))+b_{32}-B_{1}\sin(\theta_{s/u}\right)}{B_{2}\mu_{1}D_{I}}, \end{array}$$

where

5.19 $$\begin{array}{rl} D_{I} & \;=(b_{12}+B_{1}\cos(\theta_{s/u}))(b_{31}\sin(\theta_{s/u}+\psi )-b_{21}\cos(\theta_{s/u}+\psi )-B_{2})\\ & \;\;+\cos(\theta_{s/u}+\psi )+(b_{32}-B_{1}\sin(\theta_{s/u}))\sin(\theta_{s/u}+\psi ). \end{array}$$

The coefficient *A*_2_ changes sign when *D*_*I*_ changes sign and when $\cos(\theta_{s/u}+\psi )$ changes sign, the latter of which, as discussed above, occurs when the in-phase plane is crossed. The condition *D*_*I*_=0 is precisely that given in [31], p. 168 as the set of points in parameter space where the surface of saddle-node bifurcations of two mixed-mode periodic solutions intersects the in-phase cone. A positive value of *A*_2_ indicates that mixed-mode equilibria exist nearby for *μ*_2_>*μ*_20_, that is, for values of *μ*_2_ above the in-phase cone, and the opposite is true when *A*_2_<0. In conclusion, on the half of the in-phase cone where *μ*_2_ is below the in-phase plane, the stable equilibrium with *θ*=*θ*_*s*_ bifurcates supercritically when *D*_*I*_>0, so that a stable mixed-mode solution is born inside the cone, and subcritically when *D*_*I*_<0 so that an unstable (in the *r*_2_-direction) mixed-mode solution is born outside the cone. Conversely, on the half of the cone surface with *μ*_2_ above the in-phase plane, the already unstable in one direction equilibrium with *θ*=*θ*_*u*_ bifurcates supercritically when *D*_*I*_<0 giving birth to an unstable in one direction mixed-mode equilibrium outside the cone, and bifurcates subcritically when *D*_*I*_>0, giving birth to a mixed-mode solution unstable in two directions inside the cone.

Analogous results exist for the bifurcation of the anti-phase normal mode. On the half of the anti-phase cone (5.9) where *μ*_1_ is below the anti-phase plane (5.10), the bifurcation of the stable equilibrium representing the anti-phase normal mode is supercritical when

5.20 $$\begin{array}{rl} D_{A} & \;=(b_{21}+B_{2}\cos(\theta_{0}+\psi ))(b_{32}\sin(\theta_{0})-b_{12}\cos(\theta_{0})-B_{1})\\ & \;\;+\cos(\theta_{0})+(b_{31}-B_{2}\sin(\theta_{0}+\psi ))\sin(\theta_{0}) \end{array}$$

is positive, and subcritical when *D*_*A*_ is negative. On the half of the anti-phase cone surface with *μ*_1_ above the plane, the opposite is true for the unstable equilibrium with *θ*=*θ*_*u*_.

#### 5.3.2. Bifurcations at the plane

Now, consider the case where *μ*_3_ is sufficiently positive or sufficiently negative so that there are no equilibria of (4.12)–(4.14), that is $\dot{\theta }$ is never zero. As mentioned above, in this case the in-phase normal mode is represented by a periodic orbit of (4.12)–(4.14): $r_{1}=\sqrt{\mu_{1}}$, *r*_2_=0, $\dot{\theta }\neq 0$. As *μ*_2_ passes through the in-phase plane, outside the in-phase cone, this periodic orbit undergoes a pitchfork bifurcation, giving birth to two new periodic orbits (again one has *r*_2_<0 and so is unphysical) where both *r*_1_ and *r*_2_ are varying with *θ*. This new periodic orbit corresponds to quasi-periodic motion of the original system because *θ*_1_ and *θ*_2_ are varying at independent rates [31]. This solution is called a toroidal breather. In [31], we illustrated cases where this bifurcation was supercritical, and conjectured that it would be subcritical in other cases. Here, we explicitly determine the type of bifurcation, clarifying that discussion. The analysis incorporates both the bifurcation of the in-phase normal mode at the in-phase plane, and the anti-phase normal mode at the anti-phase plane.

As $\dot{\theta }$ is non-zero, we may use *τ*=*sθ*, where $s=\mathrm{sign}(\dot{\theta })$, as a time-like variable, rewriting system (4.12)–(4.14) as

5.21 $$\frac{dr_{1}}{d\tau }=\frac{r_{1}[\mu_{1}-r_{1}^{2}+(b_{12}+B_{1}\cos(s\tau ))r_{2}^{2}]}{2s[\mu_{3}+(b_{31}-B_{2}\sin(s\tau +\psi ))r_{1}^{2}+(b_{32}-B_{1}\sin(s\tau ))r_{2}^{2}]}$$

and

5.22 $$\frac{dr_{2}}{d\tau }=\frac{r_{2}[\mu_{2}+(b_{21}+B_{2}\cos(s\tau +\psi ))r_{1}^{2}-r_{2}^{2}]}{2s[\mu_{3}+(b_{31}-B_{2}\sin(s\tau +\psi ))r_{1}^{2}+(b_{32}-B_{1}\sin(s\tau ))r_{2}^{2}]}.$$

We now consider moving away from the *μ*_3_ axis a small distance *ν*>0 in a fixed direction *ξ* so that

$$\mu_{1}=\nu \cos\xi \;\text{and}\;\mu_{2}=\nu \sin\xi ,$$

and look for solutions near the trivial solution of the form

5.23 $$r_{1}^{2}=\nu R_{1}(\tau )\;\text{and}\;r_{2}^{2}=\nu R_{2}(\tau ).$$

Substituting (5.23) into system (5.21)–(5.22) yields

5.24 $$\frac{dR_{1}}{d\tau }=\frac{\nu R_{1}[\cos\xi -R_{1}+(b_{12}+B_{1}\cos(s\tau ))R_{2}]}{s[\mu_{3}+(b_{31}-B_{2}\sin(s\tau +\psi ))\nu R_{1}+(b_{32}-B_{1}\sin(s\tau ))\nu R_{2}]}$$

and

5.25 $$\frac{dR_{2}}{d\tau }=\frac{\nu R_{2}[\sin\xi +(b_{21}+B_{2}\cos(s\tau +\psi ))R_{1}-R_{2}]}{s[\mu_{3}+(b_{31}-B_{2}\sin(s\tau +\psi ))\nu R_{1}+(b_{32}-B_{1}\sin(s\tau ))\nu R_{2}]}.$$

As *ν* is small, averaging the above system over *τ*∈[0,2*π*] results in

5.26 $$\frac{dR_{1}}{d\tau }=\frac{\nu R_{1}}{\left|\mu_{3}\right|}(\cos\xi -R_{1}+b_{12}R_{2})$$

and

5.27 $$\frac{dR_{2}}{d\tau }=\frac{\nu R_{2}}{\left|\mu_{3}\right|}(\sin\xi +b_{21}R_{1}-R_{2}),$$

where we have used the fact that *s*=*sign*(*μ*_3_) for small *ν*. The equilibria for this last system are

$$(0,0),\;(\cos\xi ,0),\;(0,\sin\xi )\;\text{and}\;\left(\frac{\cos\xi +b_{12}\sin\xi }{1-b_{12}b_{21}},\frac{\sin\xi +b_{21}\cos\xi }{1-b_{12}b_{21}}\right),$$

where these are only valid if both coordinates are non-negative, because they correspond to $r_{1}^{2}$ and $r_{2}^{2}$. The Jacobian of this system is

$$J_{R}=\frac{\nu }{\left|\mu_{3}\right|}\left[\begin{array}{cc} \cos\xi -R_{1}+b_{12}R_{2}-R_{1} & b_{12}R_{1}\\ b_{21}R_{2} & \sin\xi +b_{21}R_{1}-R_{2}-R_{2} \end{array}\right].$$

Thus, at (0,0) we have

$$J_{R}(0,0)=\frac{\nu }{\left|\mu_{3}\right|}\left[\begin{array}{cc} \cos\xi & 0\\ 0 & \sin\xi \end{array}\right]=\frac{1}{\left|\mu_{3}\right|}\left[\begin{array}{cc} \mu_{1} & 0\\ 0 & \mu_{2} \end{array}\right],$$

so that the trivial solution is only stable for *ξ*∈(−*π*,−*π*/2), that is, both *μ*_1_ and *μ*_2_ negative. At the in-phase normal mode, (cos⁡ξ,0) with *ξ*∈(−*π*/2,*π*/2), so that *μ*_1_>0, we have

$$J_{R}(\cos\xi ,0)=\frac{\nu }{\left|\mu_{3}\right|}\left[\begin{array}{cc} -\cos\xi & b_{12}\cos\xi \\ 0 & \sin\xi +b_{21}\cos\xi \end{array}\right]=\frac{1}{\left|\mu_{3}\right|}\left[\begin{array}{cc} -\mu_{1} & b_{12}\mu_{1}\\ 0 & \mu_{2}+b_{21}\mu_{1} \end{array}\right],$$

which has negative eigenvalues if *μ*_2_+*b*_21_*μ*_1_<0, that is, *μ*_2_ is below the in-phase plane. At the anti-phase normal mode, (0,sin⁡ξ) with *ξ*∈(0,*π*), so that *μ*_2_>0, the Jacobian is

$$J_{R}(0,\sin\xi )=\frac{\nu }{\left|\mu_{3}\right|}\left[\begin{array}{cc} \cos\xi +b_{12}\sin\xi & 0\\ b_{21}\sin\xi & -\sin\xi \end{array}\right]=\frac{1}{\left|\mu_{3}\right|}\left[\begin{array}{cc} \mu_{1}+b_{12}\mu_{2} & 0\\ b_{21}\mu_{2} & -\mu_{2} \end{array}\right].$$

Hence, this mode is stable provided *μ*_1_+*b*_12_*μ*_2_<0, that is, *μ*_1_ is below the anti-phase plane. Finally, consider the fourth equilibrium

$$(R_{1q},R_{2q})=\left(\frac{\cos\xi +b_{12}\sin\xi }{1-b_{12}b_{21}},\frac{\sin\xi +b_{21}\cos\xi }{1-b_{12}b_{21}}\right),$$

which corresponds to the toroidal breather. This equilibrium is valid (has positive components) if *b*_12_*b*_21_<1, *μ*_1_+*b*_12_*μ*_2_>0 and *μ*_2_+*b*_21_*μ*_1_>0, or if these three inequalities are all reversed. Thus, for the breather to exist, either *b*_12_*b*_21_<1, *μ*_2_ is above the in-phase plane and *μ*_1_ is above the anti-phase plane, or *b*_12_*b*_21_>1 and *μ*_2_ and *μ*_1_ are below the in-phase and anti-phase planes, respectively. The trace and determinant of the Jacobian *J*_*R*_ evaluated at (*R*_1*q*_,*R*_2*q*_) are

$$\begin{array}{rl} \text{trace}\;[J_{R}(R_{1q},R_{2q})] & \;=-\frac{\nu }{\left|\mu_{3}\right|}(R_{1q}+R_{2q}),\\ \text{det}\;[J_{R}(R_{1q},R_{2q})] & \;={\left(\frac{\nu }{\left|\mu_{3}\right|}\right)}^{2}R_{1q}R_{2q}(1-b_{12}b_{21}). \end{array}$$

Consequently, when this breather comes into existence through bifurcation at one of the normal mode planes, it is stable if *b*_12_*b*_21_<1 and is unstable if *b*_12_*b*_21_>1.

It is important to remember that this analysis dictates the stability of solutions near the bifurcation at the in- or anti-phase plane. However, other bifurcations may exist not far away. Indeed, below we show that, for the case *b*_12_*b*_21_>1, the unstable breather solution born at the plane almost immediately collides with a pre-existing stable breather solution, annihilating each other, but this pre-existing stable breather exists for larger parameter regions on the side of the plane where the unstable breather does not exist.

## 6. Dynamics of the model applied to Huygens' clocks experiments

The analysis in §5 and [31] is here applied to the model for Huygens' clocks with parameter values appropriate for the experimental system that Huygens investigated.

### 6.1. Parameter values

The values of the system parameters that are appropriate for the case of Huygens' clocks experiments are established in this section. Huygens provided a considerable amount of information about his experiments [2,51,52], much of which is reproduced in Bennett *et al.* [3]. Based on this information, the parameter values for the model were chosen according to the assumptions and reasons given below.

(i) In Huygens' experiment, the pendula were a half pound in weight and he had placed 100 pound weights in the frames of each clock in order to help keep them stationary for sea trials. Thus, we take *m*=0.22680 kg and assume the mass of the beam to be the sum of the masses of the two weights: *M*=90.720 kg.
(ii) As Huygens used a verge escapement, the maximum displacement angle of the pendula was set to 25°, *ϕ*_max_=25(2*π*/360)=0.43633 rad.
(iii) Huygens' clocks had pendulum arms about 9 inches long and had periods very close to one second. Well-tuned pendulum clocks of his day were accurate to within about 15 s d^−1^ [32], meaning that the period of oscillation varied by less than a thousandth of a second. The length of the pendulum arm was chosen to be ℓ=0.2425 *m* (9.5472 inches) so that the period of an undamped nonlinear pendulum with maximum amplitude *ϕ*_max_ would be 1.0001 s and its angular frequency *ω*_pend_=6.2824 rad s^−1^. The corresponding angular frequency of an undamped linear pendulum with this length would be $\omega_{\mathrm{linpend}}=\sqrt{g/\ell }=6.3571\;\mathrm{rad}\;s^{-1}$.
(iv) The frequency of the uncoupled undamped beam was assumed to be about five times that of the undamped linear pendulum, that is, *ω*=5. This assumption is based on the observation that if one strikes the side of a typical table, the oscillations are clearly at a frequency much higher than one per second. We assume the beam in Huygens' experiment to behave in a similar manner. However, Senator [23] makes the opposite assumption, that the frequency of the beam is smaller than the pendula, taking the ratio, *ω*, in the range (0,0.7). He indicated that the precise value within this range was irrelevant to his results, although he only reports results for the values 0.4 and 0.7. He determined that there was a clear ‘favouring of the … pendulums-in-opposite-phase mode [and predicted] that for virtually all initial conditions, the pendulums … would synchronize [to this] steady state’ [23], p. 591. In comparison, we show below that, for Huygens' clocks, when *ω* is larger than one, the only possible stable behaviour is the anti-phase mode, and if *ω* is less than one, then mixed-mode periodic solutions, and mixed-mode 2-torus solutions, are both possible, but these solutions are themselves more anti-phase-like than in-phase. Only when *ω* becomes very small (orders of magnitude below what Senator investigated) will breather solutions and finally stable in-phase behaviour be observable.
  With our assumption of *ω*=5, the spring constant for the beam is calculated as *K*=*ω*^2^gM/ℓ=9.1655×10^4^ kg s^−2^.
(v) The beam is critically damped when $A=A_{\mathrm{crit}}=2\sqrt{MK}$, which corresponds to *β*=2*ω*. The results were not very sensitive to the precise value of *A* chosen, and so, as a default value, we chose half-critical damping: $A=\sqrt{MK}=2.8836\times 10^{3}\;\mathrm{kg}\;s^{-1}$. This value for *A* reduces the actual frequency of the beam to $\omega_{\mathrm{beam}}=(1/2M)\sqrt{4MK-A^{2}}=27.5269\;s^{-1}$, which is about 4.4 times larger than the frequency of the nonlinear undamped pendulum, *ω*_pend_. Various levels of damping, including the overdamped case, $A>2\sqrt{MK}$, are investigated below.
(vi) Huygens' clocks lost very little energy due to friction. Assuming, similar to Bennett *et al.* [3], that a 10 kg mass falling 1 m drives the clock for 3 days, the energy lost per oscillation is
  $$E_{\mathrm{lost}}=\frac{mgh}{\text{number of oscillations}}=\frac{10\;g}{60^{2}\times 24\times 3}=3.78\times 10^{-4}\;J.$$
  The maximum vertical height of the pendulum is *h*=ℓ(1−cos(*ϕ*_max_)). The vertical height lost over one cycle if there were no energy input can be computed using the energy lost per cycle via Δ*h*=*E*_lost_/(mg). The corresponding reduced angular amplitude is given by $\phi_{\mathrm{reduced}}=\arccos(1-(h-\Delta h)/\ell )$. A linear pendulum would experience an amplitude reduction over one period by a factor exp⁡(−aT/2mℓ), where *T* is the pendulum period and *a* is the damping coefficient. Thus, this coefficient may be expressed as $a=-2m\ell \log(\phi_{\mathrm{reduced}}/\phi_{\max})/T$. We take the period to be 2*π*/*ω*_pend_=1.0001 s and assume, as Senator [23], that this damping is spread equally between the air resistance and the pivot resistance, that is, *a*_*p*_=*a*_*a*_ℓ=*a*/2, thus *a*_*p*_=2.0999×10^−4^ kg m s^−1^ and $a_{a}=8.6595\times 10^{-4}\;\mathrm{kg}\;s^{-1}$.
(vii) The coefficients for the torque function, *τ*_*i*_, were chosen as follows. First, to maintain energy balance, the energy provided to the pendulum by the applied torque over one cycle was set equal to the energy lost due to friction,
  $$\Delta E=0=2\int_{-\phi_{\max}}^{\phi_{\max}}[\tau_{i}(\phi_{i},\phi_{i}^{\prime })-(a_{a}\ell +a_{p})\dot{\phi }\ell ]\;d\phi_{i}.$$
  An analytic solution to the above integral can be obtained by approximating the trajectory of the pendulum with that of a linear undamped pendulum: $\phi (\hat{t})=\phi_{\max}\sin\hat{t}$. This yields $0=\pi \phi_{\max}^{2}(b-c\phi_{\max}^{2}/8-(a_{a}\ell +a_{p})\sqrt{lg})$, hence *c* is given in terms of *b* by
  6.1 $$c=\frac{8}{\phi_{\max}^{2}}(b-(a_{a}\ell +a_{p})\sqrt{\ell g}).$$
  Although this is just an approximation, even at values of *ϕ*_max_ near 25°, it is still quite good because the damping is very small. So, for *c* given by (6.1), the actual maximum amplitude of a single uncoupled pendulum is very close to the specified *ϕ*_max_ value. In terms of the non-dimensional parameters this relation is *γ*=16*δ*/*ϕ*^2^_max_.
   Second, the value of *b* was constrained so that the torque exceeded the friction near the origin, that is, *δ* was positive, otherwise the origin would be stable, and so that the torque always added energy, that is, was always the same sign as *ϕ*′. Again, using $\phi (\hat{t})\approx \phi_{\max}\sin\hat{t}$, the torque is the same sign as *ϕ*′ when
  $$\phi_{\max}\leq \sqrt{\frac{2b}{c}}.$$
  These two constraints along with the energy balance, (6.1), force *b* to satisfy
  $$(a_{a}\ell +a_{p})\sqrt{\ell g}<b<\frac{4}{3}(a_{a}\ell +a_{p})\sqrt{\ell g}.$$
  Using the physical values established so far, we obtain the following numerical ranges for *b* and *c*:
  6.2 $$6.4744\times 10^{-4}<b<8.6325\times 10^{-4}$$
  and
  6.3 $$0<c<9.0686\times 10^{-3},$$
  where the units for *b* and *c* are kg m^2^ s^−2^. The specific value of *c* within its range is dictated by the choice of *b* through (6.1). As a default value, we chose *b*=8.1190×10^−4^ kg m^2^ s^−2^, which makes the linear energy input to the system about 25% larger than the loss due to friction. The corresponding default value for *c* is 6.9103×10^−3^ kg m^2^ s^−2^. We tested other values of *b* (and *c*) in these ranges, but there were no significant changes in the results.
(viii) For Huygens' clocks, we set *d*=*k*=0 so that there is no direct coupling between the pendula.

Using the above values for the physical constants for the verge escapement, the non-dimensional parameters, given by (2.7) are

6.4 $$\begin{array}{rl} \delta & \;=1.526\times 10^{-4},\;\epsilon =2.500\times 10^{-3},\;\eta =0,\;\kappa =0,\\ \alpha & \;=6.006\times 10^{-4},\;\beta =5.000,\;\gamma =1.282\times 10^{-2}\;\text{and}\;\omega^{2}=25.00. \end{array}\}$$

The parameters in the normal form may be calculated from the above using (4.4), (4.5) and (4.20)–(4.25) giving

6.5 $$\begin{array}{rl} \mu_{1} & \;=1.3170\times 10^{-4},\;\mu_{2}=1.5256\times 10^{-4},\;\mu_{3}=-9.9821\times 10^{-5},\;\psi =3.1161,\\ b_{12} & \;=-1.9733,\;b_{21}=-2.0267,\;B_{1}=77.949,\;B_{2}=79.012,\\ b_{31} & \;=79.060\;\text{and}\;b_{32}=-77.888. \end{array}\}$$

Several important consequences on the geometry of the bifurcation surfaces in parameter space and the location of the Huygens' clocks system in parameter space follow from these values. First, note that *μ*_1_<*μ*_2_ and *μ*_3_<0. The fact that this will be the case follows from our physical assumptions that the air resistance, *α*, is small, that the frequency of the beam is higher than that of the pendula: *ω*>1, and that there is no direct coupling between the pendula (*η*=*κ*=0). Because of these assumptions, from (4.4) it follows that *C*_1_ and *C*_2_ are positive, and then from (4.20), as *ϵ*, the mass ratio, is positive, it follows that *μ*_1_<*μ*_2_ and *μ*_3_<0. Second, as *b*_12_*b*_21_≈4>1, the toroidal breather solution that comes from *μ*_1_ (*μ*_2_) decreasing across the in-phase (anti-phase) plane is initially unstable. Third, as *B*_1_≈*B*_2_, the cones are almost the same size. Fourth, since 0<*b*_31_≈*B*_2_, the *μ*_3_ coordinate of the centre line of the in-phase cone decreases with increasing *μ*_1_ at the same rate as the radius of the cone expands; further, this expansion is quite rapid as *B*_2_ is large. The *μ*_2_ coordinate of the centre line of the in-phase cone shifts at a much slower rate because |*b*_21_|≪*b*_31_. Similarly, the *μ*_3_ coordinate of the centre line of the anti-phase cone increases with increasing *μ*_2_ at the same rate as the radius of that cone expands; this expansion is rapid, and the *μ*_1_ coordinate of the centre line shifts slowly. Consequently, the interior of the in-phase cone fills almost all of the region *μ*_1_>0, *μ*_3_<0, and the interior of the anti-phase cone fills almost all of the region *μ*_2_>0, *μ*_3_>0, that is, each about a quarter (24%) of the parameter space.

If one ignores the contributions of *δ*, *ϵ*, *η* and *κ* to the parameters of the higher-order terms, as in the specific normal form (4.26)–(4.28), their values become

6.6 ψ =2 arctan ⁡(1γ)=3.1160,b12 =b21=−2B1 =B2=1+γ2γ=78.004andb31 =−b32=1γ=77.998.}

The changes in these values compared to (6.5) are not significant in terms of the geometry of parameter space. The fact that there is exact equality: *B*_1_=*B*_2_, *b*_12_=*b*_21_ and *b*_31_=−*b*_32_, when the contributions of the small parameters are ignored implies that (i) system (4.12)–(4.14) has an additional symmetry, being invariant under the transformation (*μ*_1_,*μ*_2_,*μ*_3_,*r*_1_,*r*_2_,*θ*)↦(*μ*_2_,*μ*_1_,−*μ*_3_,*r*_2_,*r*_1_,−*θ*−*ψ*) and (ii) the in-phase and anti-phase cones intersect tangentially along the line *μ*_1_=*μ*_2_, *μ*_3_=0. When the small parameters are taken into account, this additional symmetry is broken, and the tangential intersection of the cones splits into two nearby transverse intersections.

### 6.2. Bifurcations and stable solutions

**Figure 7.**
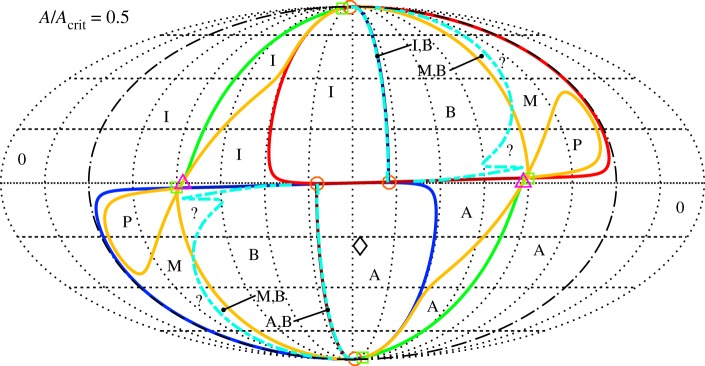
Bifurcation diagram for Huygens' clocks system (4.12)–(4.14) and the parameters given by (6.6). The figure shows a Mollweide projection of a sphere centred at the origin in *μ*-space with central meridian the plane *μ*_1_=*μ*_2_>0. The in-phase (lower left, blue) and anti-phase (upper right, red) cones each enclose almost a quarter of the space. Most of the in-phase plane (all that is discernible with this resolution) lies inside the anti-phase cone and vice versa. The surfaces of saddle-node bifurcations of mixed-mode equilibria (green) are the most exterior solid lines in the upper left and lower right. The phantom bifurcation curves are not shown since they are almost coincident with the in-phase and anti-phase cones. The (black) dashed curve separating regions labelled 0 and I is the primary pitchfork bifurcation of the origin giving rise to the stable in-phase normal mode solution. Similarly, the (black) dashed curve separating regions 0 and A is the pitchfork bifurcation giving rise to the stable anti-phase normal mode. Interior (orange) curves are Hopf bifurcations of the mixed-mode equilibria. The (magenta) triangles mark Fold-Hopf bifurcations where the surface of saddle-node bifurcations intersect with the Hopf curves. The dot-dashed curves (light blue) mark where stable periodic breather orbits of the 3D system, which have unbounded *θ* values, lose stability in some manner. The labels indicate the stable solutions present in each region, 0, origin equilibrium; I, in-phase normal mode; A, anti-phase normal mode; M, mixed-mode periodic solution; P, mixed-mode 2-torus solution with bounded *θ* values; B, toroidal breather solution (unbounded *θ* values); ?, more complicated solutions. The (black) diamond shows the location of *μ* for our model of Huygens' clocks. Other symbols as in figure 6.

The bifurcation diagram for system (4.12)–(4.14) using the parameter values given by (6.6) is shown in figure 7; the additional symmetry is clearly evident in this Mollweide map as a reflection through the origin. As mentioned above, the cones are very large, each occupying about one-quarter of the parameter space, hence they no longer appear circular on the Mollweide map, and they intersect tangentially at *μ*_1_=*μ*_2_, *μ*_3_=0 (the centre of the diagram). As the product *b*_12_*b*_21_ is greater than one, and both these parameters are negative, the in-phase plane exists to the right of centre, and the anti-phase plane to the left. The in-phase plane lies almost completely inside the anti-phase cone and vice versa. As discussed in §5.3, the values of *D*_*I*_ and *D*_*A*_, (5.19) and (5.20), dictate the super- or subcriticality of the pitchfork bifurcations on the cone surfaces. For the parameter values given by (6.6), these simplify to

6.7 $$D_{I}=\frac{\sqrt{1+\gamma^{2}}}{\gamma }\left(1-\cos\left(\theta +\frac{\phi }{2}\right)\right)\left[\gamma \left(1-3\cos\left(\theta +\frac{\phi }{2}\right)\right)-\sin\left(\theta +\frac{\phi }{2}\right)\right]$$

and

6.8 $$D_{A}=\frac{\sqrt{1+\gamma^{2}}}{\gamma }\left(1-\cos\left(\theta +\frac{\phi }{2}\right)\right)\left[\gamma \left(1-3\cos\left(\theta +\frac{\phi }{2}\right)\right)+\sin\left(\theta +\frac{\phi }{2}\right)\right].$$

Clearly, *D*_*I*_ and *D*_*A*_ can only change sign with varying *θ* when the expressions inside the square brackets, respectively, change sign, which can be shown to occur exactly twice on [0,2*π*]. (The zeros of *D*_*I*_ and *D*_*A*_ that occur at *θ*=−*ϕ*/2 are second order in *θ* and correspond to the location where the two cones tangentially intersect; however, the type of bifurcation on both sides of this point is unchanged because *D* does not cross zero.) Thus, moving clockwise around the in-phase cone curve in figure 7, the pitchfork bifurcation on the cone surface is supercritical starting from the double zero bifurcation (circle) near the south pole, becomes subcritical where the upper surface of saddle node bifurcations meets it at the square near the equator, becomes supercritical again when it passes the intersection with the in-phase plane at the centre of the diagram and then becomes subcritical again for a short time after it passes the intersection with the lower saddle node surface near the south pole. The anti-phase cone bifurcations behave analogously.

Besides the in- and anti-phase cones and planes and the surface of saddle-node bifurcations of mixed-mode equilibria, the diagram also shows other bifurcation curves (numerically generated) and labels different regions to indicate the stable solutions present within each. In the region labelled 0 (*μ*_1_<0 and *μ*_2_<0), which appears on the far left and right of the diagram, the equilibrium at the origin is the only stable solution. The dashed curve on the left, separating regions 0 and I, is the primary pitchfork bifurcation of the origin giving rise to the stable in-phase normal mode solution. In the northern hemisphere, as one moves east, this in-phase normal mode remains the only stable solution, despite crossing other bifurcation curves that give rise to other (unstable) solutions. The first of these curves crossed as one moves east, is the (green) solid line marking the saddle-node bifurcation of mixed-mode phase-locked equilibria. To the right of this curve there are two such equilibria, one with a 2D unstable manifold and the other with a 3D unstable manifold. The next bifurcation crossed is the (orange) curve marking a Hopf bifurcation of the second of these mixed-mode equilibria. Even though the equilibrium gains two stable dimensions, it is still unstable in the third dimension and the associated periodic orbit is also unstable in at least one dimension. Next, just before the anti-phase cone (solid red) is crossed, the anti-phase normal mode is born (unstable in two dimensions) in a pitchfork bifurcation of the origin along the line *μ*_2_=0. This line is not marked specially on the diagram, but is the dotted line of longitude zero, which is the second one to the left of centre, nearly coincident with the anti-phase cone. When the anti-phase cone is crossed, a subcritical pitchfork bifurcation absorbs one of the mixed-mode equilibria and reduces the unstable dimension of the anti-phase normal mode to one. Still at this point, the in-phase normal mode is the only stable solution. This remains the case as one moves further east until just before the in-phase plane is reached. At this point, a saddle node of cycles bifurcation is crossed (light blue dot-dashed) where a pair of breather solutions is born, one stable and one unstable. The region labelled I,B, where both the one breather solution and the in-phase mode are stable, is extremely narrow. As the in-phase plane is crossed on the right edge of this region, the unstable breather is absorbed into the in-phase mode in a pitchfork of cycles, making the in-phase mode unstable, and leaving the other breather orbit as the only stable solution (region B). This situation persists as one moves further east for a considerable distance. In the far north, the next curve crossed is the (orange) Hopf bifurcation of the remaining mixed-mode equilibrium. This bifurcation is subcritical and to its right the mixed-mode equilibrium is stable, surrounded by an unstable limit cycle. Thus, in the region labelled M,B there are two stable solutions, the breather and the mixed-mode periodic solution. On the east border of this region (light blue, dot-dashed curve), the synchronized breather solution loses stability in a period doubling bifurcation. The stable double-period solution exists for a short span but then bifurcates into other more exotic solutions in the region marked ‘?’, until the mixed-mode solution becomes the only stable solution in the region M. Figure 5 shows the period doubling sequence leading from a synchronized breather through to a chaotic breather solution. Heading further east, the mixed-mode solution is absorbed in a supercritical pitchfork bifurcation on the surface of the anti-phase cone making the anti-phase normal mode the only stable solution, but almost immediately the curve at *μ*_1_=0 (black, dashed) is crossed where the anti-phase normal mode disappears into the origin in the primary pitchfork bifurcation and we are back in region 0.

Returning to region B, if one moves east from region B while remaining close to the equator, the situation is a little different. Here, before the Hopf bifurcation of the mixed-mode equilibrium is reached, the breather solution loses stability (light blue, dot-dashed curve) and the region labelled ‘?’ is entered where it appears numerically that chaotic solutions exist. On part of this boundary, the bifurcation is a period-doubling one, but below the switch-back, the loss of stability of the breather appears to be due to a homoclinic bifurcation where the breather meets one of the two unstable anti-phase equilibria (*r*_1_=0, $r_{2}=\sqrt{\mu_{2}}$, *θ*=*θ*_s/u_) and gives birth to a stable periodic orbit with bounded *θ* values, which, as one continues to the east, eventually dies at the Hopf bifurcation curve of the mixed-mode equilibrium, which here is supercritical. Thus, we enter region M with only the mixed-mode equilibrium being stable. There is another Hopf bifurcation curve surrounding the region labelled P. This curve, although it appears at the resolution of the diagram to intersect some of the other curves, is in fact an isolated simple closed loop. Everywhere on this loop, the Hopf bifurcation of the mixed-mode equilibrium appears to be supercritical so that in the region P the mixed-mode equilibrium is unstable, but there is a stable periodic solution with bounded *θ* values, that is, a mixed-mode 2-torus solution. Just south-west of region P, the curve of saddle-node bifurcations of the mixed-mode equilibria intersect with the anti-phase cone (marked as a square on the diagram), and the curve of Hopf bifurcations on the west side of region M meets the saddle-node curve at a Fold-Hopf bifurcation marked by a triangle. Near these points more complicated phenomena occur, including a tiny region where there is bi-stability with a mixed-mode solution and the anti-phase normal mode.

An analogous set of transitions occurs as one moves from region 0 westwards in the southern hemisphere, but here the in-phase and anti-phase roles are switched. The regions marked A have only the anti-phase normal mode as a stable solution.

The above description covers the main qualitative changes of behaviour of solutions. A more thorough investigation could be done especially regarding the Hopf bifurcation curves and the bifurcations where the stable breather solution loses stability; however, our focus here is in another region of the parameter space.

In figure 7, the diamond marks the location of the *μ* values corresponding to the values of the physical constants chosen for our model of Huygens' clocks. As can be seen from the figure, the point is well inside the region where only the anti-phase normal mode is stable; consequently it is not surprising that Huygens observed this phenomenon only. Further, from (4.20) and (4.4) it is evident that with small *α* (pendulum damping), as long as *ω*>1 (the beam frequency is larger than the pendulum frequency), we will have *μ*_1_<*μ*_2_, *μ*_2_>0 and *μ*_3_<0 so that the system lies somewhere in the lower right quarter of the bifurcation diagram where the anti-phase normal mode is the only stable solution. In the next subsection, alterations are made to the chosen physical parameters to investigate when it is possible to see other stable solutions in Huygens' clocks system.

### 6.3. Possibility of observing other stable solutions for Huygens' clocks

In this section, it is shown that anti-phase normal mode behaviour is the predominant stable mode in Huygens' clocks system unless one extremely deviates away from the physical set-up that Huygens was using.

The three physical parameters that are altered in this section are the beam damping, *A*, the beam restoring force constant, *K* and the cubic torque coefficient, *c*. The masses *m* and *M* are not altered as the analysis is dependent on *ϵ*=*m*/*M*≪1. The other physical parameters (ℓ, *g*, *a*_*a*_, *a*_*p*_, *b*) are held at their default values given in §6.1. Varying *A* and *K* causes alterations in the non-dimensional parameters *β* and *ω*, (2.7), respectively. The beam damping is allowed to vary from near zero to two times critical damping. The beam restoring force, *K*, is allowed to vary so that the frequency ratio, $\omega =\sqrt{\ell K/gM}$, varies between 10^−3^ and 6. The difference between a verge escapement and an anchor escapement is investigated by setting *ϕ*_max_ at either 25° or 8° (0.43633 or 0.13963 radians). Changing *ϕ*_max_ alters the value of the torque coefficient *c* through (6.1) and hence the non-dimensional parameter *γ*. Given that the pendulum friction coefficients are unchanged, the default value for *c* for an anchor escapement is 1.1997×10^−1^ kg m^2^ s^−2^, and this increase in *c* compared to a verge escapement value of 6.9103×10^−3^, has a significant impact on the results as discussed below.

As *A* and *K* vary, the values of all the normal form parameters in system (4.12)–(4.14), particularly *B*_1_, *B*_2_, *b*_31_ and *b*_32_ will vary. This alters the cones and planes (and other bifurcation curves) in the bifurcation diagram somewhat, but for the most part their relative locations remain the same. Varying *A* has the smallest effect on the geometry of the planes and cones, while *ω* has a large effect but primarily when it is near 1, that is, when the system is near resonance. Indeed, for all values of *A* and *K* that are being considered, for the verge escapement the relations

$$b_{31}\approx B_{2}\;\text{and}\;b_{32}\approx -B_{1}$$

continue to hold so that the cones' radii grow roughly equally with the vertical movement of their centre lines. Further, the in-phase cone is primarily in the lower hemisphere (*μ*_3_<0) and the anti-phase cone is primarily in the upper hemisphere.

**Figure 8.**
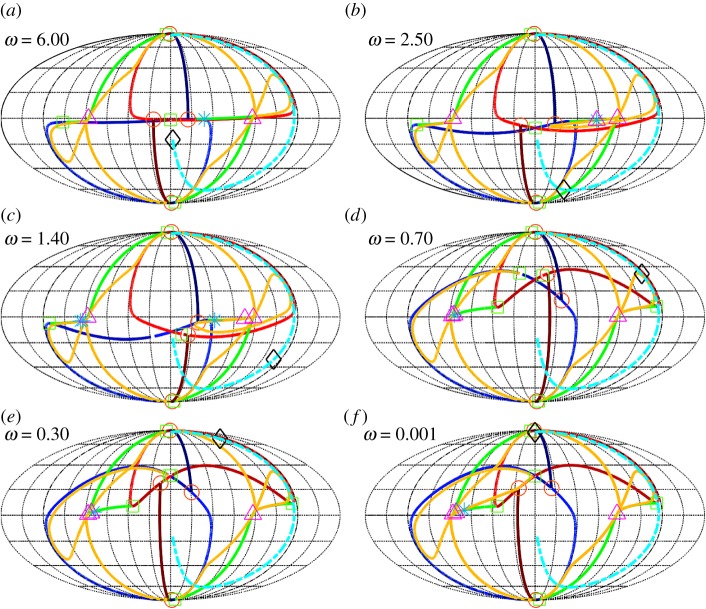
Bifurcation diagrams for system (4.12)–(4.14) with verge escapement at various values of the beam to pendulum natural frequency ratio, *ω* as specified. The (light blue) dotted-dashed curve shows the track of the system as *ω* varies, with the diamond indicating the location on the track for the current value of *ω*. Other symbols and colours are as in figures 6 and 7.

Figure 8 illustrates the changes in the bifurcation diagram in *μ*-space as *ω* varies while beam damping remains at half critical. For each value of *ω* indicated in the figure, the bifurcation diagram is plotted and the system location is given by the (black) diamond. The (light blue) dot-dashed curve shown on all plots traces out the path of this diamond as *ω* decreases from 6 to 0.01. As seen in this figure, as *ω* decreases, the system first moves south and east, crossing to the exterior of the in-phase cone but on the side where only the anti-phase mode is stable (figure 8*a,b*). After passing near the south pole, the system moves northwards just west of the *μ*_2_=0 meridian (figure 8*c*). As *ω* decreases past $\frac{1}{2}(\alpha +\sqrt{\alpha^{2}+4})$, where *C*_2_ is zero, the system enters the northern hemisphere, that is, *μ*_3_>0. Shortly thereafter (at about *ω*=0.938), the system crosses into the interior of the anti-phase cone where now, for the first time, the anti-phase mode is unstable. The mixed-mode solution is stable in this region, although, in terms of the oscillations of the two physical clocks, there is no abrupt change in behaviour as only a very small amount of the in-phase mode is present because the system is just barely inside the anti-phase cone. As *ω* decreases further, the system moves northwards and skirts along the Hopf bifurcation curve, crossing into its interior for a short interval *ω*≈[0.748,0.790] (figure 8*d*). In this small interval of *ω* values, the mixed-mode 2-torus solution (P) is stable, where the mixed-mode periodic solution has given birth to a limit cycle with bounded *θ* values. However, the system is just barely across this curve, so the limit cycle is very small and the physical manifestation would probably appear as noise on top of the mixed-mode periodic solution. As *ω* continues to decrease, the system moves almost directly northwards, staying very close to and just west of the *μ*_2_=0 meridian (figure 8*e*). The system finally crosses the in-phase plane very near the north pole (at about *ω*=0.031) at which point the in-phase mode becomes the stable attracting solution (figure 8*f*). In conclusion then, simply reducing the restoring force of the beam is insufficient to produce stable in-phase motion until the restoring force is very small, so that the ratio of the natural frequency of the beam to the pendula is about 0.03.

**Figure 9.**
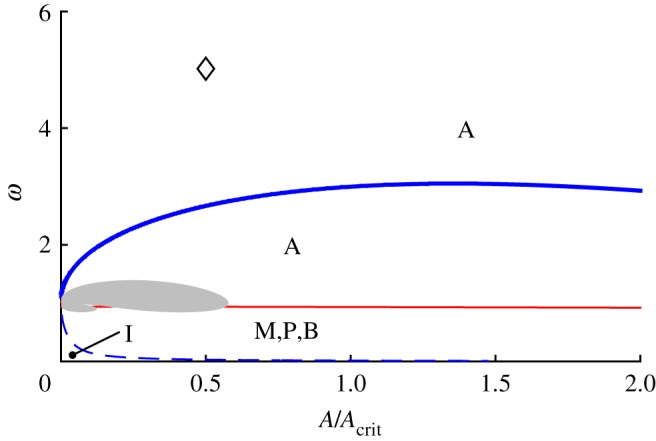
Bifurcation diagram in terms of the non-dimensional parameters *A*/*A*_crit_ and *ω* for Huygens' clocks system (4.12)–(4.14) with verge escapement. The thick solid (blue) line is the in-phase cone; the thin solid (red) line is the anti-phase cone; the dashed (blue) line is the in-phase plane; the diamond is the location for the default parameters for Huygens' clocks system. The anti-phase plane is not located within the ranges of the parameters considered here. The labels indicate stable solutions present in each region: I, in-phase normal mode; A, anti-phase normal mode; M, mixed-mode periodic solution; P, mixed-mode 2-torus solution (bounded *θ* values); B, toroidal breather solution (unbounded *θ* values). Boundaries between M, P and B solutions were not computed; in part of that region multiple modes are simultaneously stable. The greyed-out region is the near-resonance region where *ω* is too close to one and the beam damping too small for the analysis in this paper to be valid.

Figure 9 shows a 2D bifurcation diagram (cones and planes only) for the parameter pair (*A*/*A*_crit_,*ω*). In this plot, a portion of the in-phase cone appears as the thick (blue) solid line, but on either side of it only the anti-phase mode is stable. The anti-phase cone appears as the thin solid (red) line very near *ω*=1. Once it is crossed, the mixed-mode periodic solution becomes the stable solution, and then later, as *ω* is decreased more, the breather solution is the stable attractor. In parts of this region, both the mixed-mode periodic solution and the breather are simultaneously stable. Mixed-mode 2-torus solutions with bounded *θ* values also exist in part of this region, although only in a very narrow sliver of *ω* values around *ω*=0.7. The in-phase plane appears as the dashed curve in the bottom left corner of the diagram. Only in this very small region below the dashed curve, where the beam damping is very small and the beam to pendulum frequency ratio is below one, is the in-phase normal mode the stable attractor.

**Figure 10.**
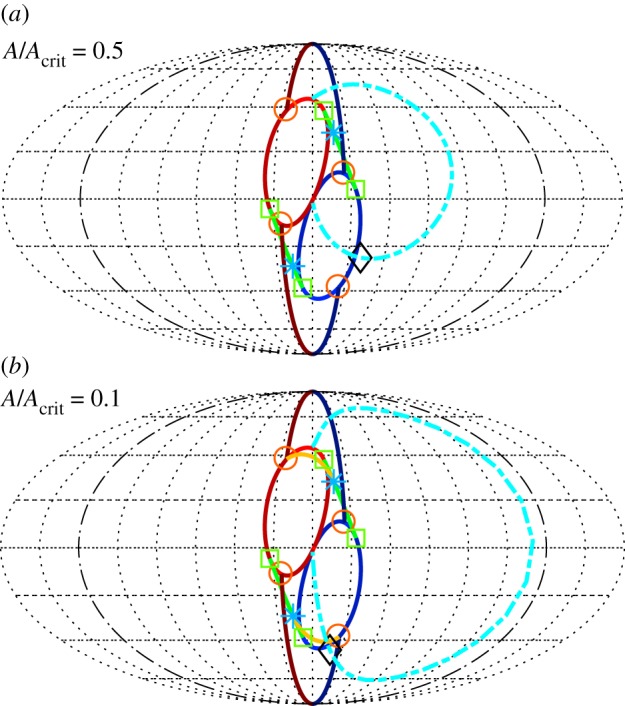
Bifurcation diagrams for system (4.12)–(4.14) with anchor escapement at the values of *A*/*A*_crit_ as specified. The (light blue) dot-dash curve shows the track of the system as *ω* varies, with the diamond indicating the location on the track for *ω*=1.45. Symbols and colours as in figure 8.

**Figure 11.**
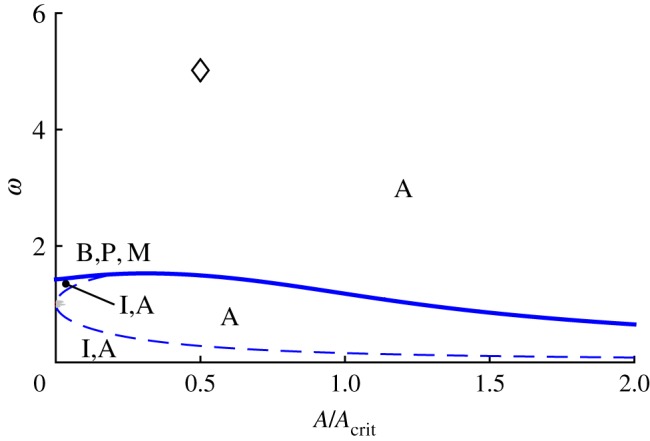
Bifurcation diagram in terms of the non-dimensional parameters *A*/*A*_crit_ and *ω* for Huygens' clocks system (4.12)–(4.14) with anchor escapement. Symbols and colours as in figure 9.

By contrast, when one models the anchor escapement, using *ϕ*_max_=8°, the bifurcation diagrams become those shown in figures 10 and 11. Noticeably, the cones in figure 10 are much smaller than those in figure 8, and the anti-phase cone has moved to the left of the in-phase cone, although the anti- and in-phase planes are still oriented the same way. These effects are due to the decrease in *ϕ*_max_, which, through (6.1), causes a large increase in *c* and consequently an increase in *γ*. Therefore, the cone sizes, dictated by *B*_1_ and *B*_2_, are smaller (4.22)–(4.23). In addition, with this large value of *γ*, the values of the non-dimensional parameters *b*_12_, *b*_21_, *b*_31_, *b*_32_, *B*_1_ and *B*_2_ show almost no change as *ω* and *β* vary. For this reason, the locations of the planes and cones do not change as *ω* varies at the scale of the diagrams, so a single diagram is sufficient for all values of *ω*. (The curves in figure 10 are for *ω*=1.45.) Figure 10*a* shows the track of the system as *ω* decreases from 6 to 0.001 as the dot-dash (light blue) curve using the default value of *A*/*A*_crit_=0.5 (half-critical beam damping). In this case, the system starts inside the in-phase cone and then exits the right side of the cone, but again only the anti-phase mode is stable in both areas. Eventually, at about *ω*=0.46 the system crosses the in-phase plane in the northern hemisphere to a region where both the in-phase and anti-phase modes are stable. The situation is more interesting if *A*/*A*_crit_ is reduced to 0.1 (10% of critical damping) as illustrated in figure 10*b*. Here, although the planes and cones have not moved appreciably, the values of *μ* are different due to the smaller value of *β*, see (4.20). Now, as *ω* decreases from 6.0, the system first is in a region where the anti-phase mode alone is stable, then briefly enters a region where breather, mixed-mode 2-torus solutions and then mixed-mode periodic solutions are in turn simultaneously stable with the anti-phase mode. At about *ω*=1.48, the system exits the in-phase cone on the *left* of the in-phase plane into a region between the planes where both the in-phase and anti-phase modes are stable. The black diamond in the figure shows the location of the system in this region at *ω*=1.45. As *ω* continues to decrease, the in-phase plane is crossed at about *ω*=1.28, leaving only the anti-phase mode stable, which remains the case until about *ω*=0.72 where the system again crosses to the left of the in-phase plane to the region in the northern hemisphere where both the normal modes are stable. The bifurcation diagram in terms of the parameters *A*/*A*_crit_ and *ω* is shown in figure 11. This figure should be compared with figure 9. Whereas using parameter values relevant for a verge escapement, figure 9, the anti-phase mode is the only stable mode unless *ω* and *A*/*A*_crit_ are very small, the situation when using parameter values relevant for an anchor escapement, figure 11, admits regions where both in-phase and anti-phase modes are simultaneously stable including a region where *ω*>1 when *A*/*A*_crit_ is small.

### 6.4. Relation to other studies

**Figure 12.**
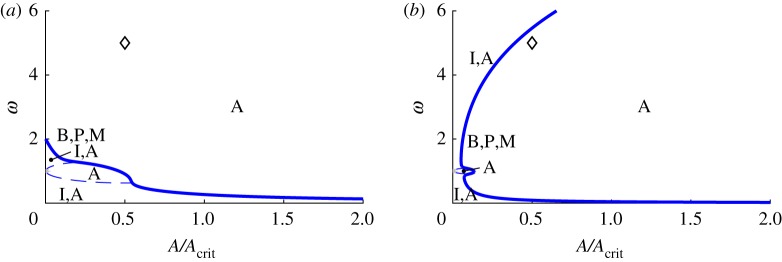
Bifurcation diagram in terms of the non-dimensional parameters *A*/*A*_crit_ and *ω* for Huygens' clocks system (4.12)–(4.14) with anchor escapement and maximum displacement angle 5° (*a*) and 3° (*b*). Symbols and colours as in figure 9.

Blekhman analyses Huygens' problem and reports that ‘regardless of the ratio between the pendulum frequency and the natural frequency of the platform, both synphaseous and antiphaseous motions of the pendulums are stable’ [10], p. 154. He also reports that both in-phase and anti-phase solutions were observed in experiments done at the Mekhanobr Institute. The important assumptions in his analysis are that the angular deflections of the pendula are small and the viscous damping of the beam is small [10], p. 148. This corresponds to the left edge of figure 11. Here, using a maximum displacement angle of 8°, the two normal modes are simultaneously stable for *ω*<1.6. However, if displacement angles are reduced further as shown in figure 12, then the region at low values of damping where both normal modes are simultaneously stable extends to higher values of the beam to pendulum frequency ratio, *ω*, and indeed, if the maximum angle is below 3°, the region of dual stability encompasses all values of *ω* when *A*/*A*_crit_ is small (figure 12*b*). As Blekhman's analysis is for small angular displacement and small beam damping, the relevant bifurcation diagram would be similar to that of figure 12*b*, where clearly both in-phase and anti-phase modes are stable for all *ω* when *A*/*A*_crit_ is small, agreeing with his conclusions.

The experimental system of Bennett *et al.* [3] was two clocks with anchor escapements on a cart running on a low-friction track. The cart had no restoring force and so their system corresponds to ω→0. Their ratio of masses was larger than those analysed here, being in the range 0.0064≤*ϵ*≤0.0128, whereas we have *ϵ*=0.0025. Their main finding was that, at lower values of *ϵ*, only the anti-phase motion is stable, but at higher values ‘beating death’ occurred, where one pendulum ceased operating because it had passed too much energy to its neighbour and found itself below the escapement threshold. It is only the mixed-mode 2-torus solutions and the toroidal breather solutions that exchange energy between the in-phase and anti-phase modes, with the breather solution having typically more energy exchange since the *θ* values are unbounded, so we conclude that they were probably witnessing breather solutions, which then collapsed when one pendulum no longer had sufficient amplitude to engage the escapement mechanism.

Pantaleone studied an experimental system with pendulum metronomes resting on a base free to roll on empty pop cans [22]. This system again has no restoring force for the support and very small friction for the movement of the base, and so, in terms of the present analysis, the limits ω→0 and A→0 are appropriate. He also mainly studied the case where the mass of the support was not very large, which is outside the analysis of the present study because it means *ϵ* is not small. His finding was that his standard system always exhibited in-phase motion, and even when mass was added to the base (*ϵ* is now small), in-phase motion remained the only stable solution. He attributed the lack of anti-phase synchronization to the fact the metronomes had very large amplitudes of oscillation, about 45°. However, as seen in figure 9, which is for a maximum amplitude of 25°, relevant for Huygens' system, we see that, for small *ω* and *A*, the only stable solution is the in-phase motion, in complete agreement with Pantaleone's findings. Indeed, the bifurcation diagram (not shown) for the case with maximum amplitude 45° is not much altered from that shown in figure 9. Thus, the reason that Pantaleone's system only exhibited in-phase solutions was not due to the large amplitudes of the oscillations but rather due to the lack of a restoring force for the base. Pantaleone also reports that if water was put on the surface, which increased the friction of the rolling pop cans, then near anti-phase motion was observed. This corresponds to increasing *A*/*A*_crit_ in figure 9 while keeping *ω* close to zero. It can be seen that this adjustment would move the system out of the region where the in-phase mode is stable into the region where mixed-mode periodic solutions, breathers and/or mixed-mode 2-torus solutions are stable. The figure is not quite applicable to Pantaleone's case though, because the mass on the bases had been removed, making *ϵ* large again. Nonetheless, we suspect that the equivalent bifurcation diagram relevant for large *ϵ* would look similar to figure 9 at least in the region of small *ω*, and so suspect he was observing a mixed-mode periodic solution that was dominated by the anti-phase normal mode and consequently was phase-locked near a 180° difference.

The analysis of Senator [23], like Bennett *et al.* [3], also concludes that the anti-phase mode is the favoured mode. His analysis included a 5-degrees of freedom model where the clock cases were independent of the clocks themselves. This more complicated model did not provide any different results, justifying the conclusions of Blekhman and others that the 3-degrees of freedom model, like that used in this manuscript, is sufficient to explain Huygens' findings.

Stable mixed-mode periodic solutions were also reported in Belykh *et al.* [25] and Czolczyński *et al.* [53], although in the latter study mixed-mode periodic solutions were only found in the case where the two pendulum masses were different.

## 7. Conclusion

The analysis presented here provides a detailed description of the behaviour of a system with two weakly coupled identical oscillators near the onset of oscillations from the rest state due to a double Hopf bifurcation. This was achieved through the application of equivariant bifurcation and normal form theory applied to a system with ‘permutation’ symmetry. In addition, the computations were made to identify the precise dependence of the normal form parameters on the physical system parameters. This allows for the classification of the type of solution expected for a physical system with a specific set of physical parameter values.

The types of behaviour for the system near the double Hopf bifurcation are the rest state, the well-known in- and anti-phase periodic motions, mixed-mode periodic solutions, mixed-mode 2-torus solutions with bounded values in the mode phase difference (arising from secondary Hopf bifurcations of a mixed-mode periodic solution), breather solutions, with mode phase difference values continually wrapping (modulo 2*π*), and chaotic breather solutions arising from a period-doubling cascade of a breather solution. It is the latter three solutions, and particularly the breathers, that exchange energy between the two oscillators.

The mapping of the physical system parameters to the normal form parameters shows that Huygens' experimental system (two identical pendulum clocks attached to a common beam) lies well inside a region where only anti-phase motion is stable, explaining why this was the only motion he observed. The analysis is valid provided the difference between the energy input and the energy lost due to friction, *δ*, is small, and the ratio of the pendulum bob mass to the beam mass, *ϵ*, is small. In addition, it was shown that substantial deviations away from Huygens' physical set-up must be made in order for in-phase or other solutions to be stable. Deviations that result in stable in-phase solutions include making the ratio of the beam frequency to the pendulum frequency very small (*ω*≪1), as would be the case with very small restoring force applied to the beam. Ratios closer to *ω*=1 can also give stable in-phase solutions provided the damping of the beam is not too large, and the maximum amplitude of the oscillators is sufficiently small. It was shown that clocks using anchor escapements, which have maximum pendulum amplitudes around 8°, could exhibit stable in-phase motion when *ω* was about 1.5 and the beam damping was sufficiently small, but that clocks using verge escapements, like the clocks used by Huygens, which needed amplitudes near 25°, exhibited only anti-phase motion.

The model presented here uses a continuous representation of the escapement mechanism that continues to engage, unlike real clocks, even for very small oscillation amplitudes. Thus, the model cannot replicate the ‘beating death’ phenomenon observed by Bennett *et al.* [3] where one pendulum comes to rest due to losing too much energy to the other and being unable to engage its escapement. Breather solutions, which exchange energy between the pendula, are stable in parameter regions attainable by some experimental set-ups. A system with an escapement threshold has the potential of exhibiting beating death when in this region because the breather solution may exchange enough energy between the two pendula to cause one to drop below the threshold.

## Data accessibility

The videos of the breather solutions mentioned in §5 are available from the Dryad Digital Repository: http://dx.doi.org/10.5061/dryad.p4sg5 [42].

## References

[1] Huygens C 1669 Instructions concerning the use of pendulum-watches for finding the longitude at sea. *Phil. Trans.* 4, 937–976. (doi:10.1098/rstl.1669.0013)

[2] Huygens C 1893 *Oeuvres complètes de Christiaan Huygens*, vol. 5 The Hague, The Netherlands: Martinus Nijhoff Includes correspondence from 1665.

[3] Bennett M, Schatz MF, Rockwood H, Wiesenfeld K 2002 Huygens's clocks. *Proc. R. Soc. Lond. A* 458, 563–579. (doi:10.1098/rspa.2001.0888)

[4] Czolczyński K, Perlikowski P, Stefański A, Kapitaniak T 2011 Huygens' odd sympathy experiment revisited. *Intl. J. Bifurcation Chaos* 21, 2047–2056. (doi:10.1142/S0218127411029628)

[5] Korteweg DJ 1906 Les horloges sympathiques de Huygens. *Arch. Neerlandaises II* XI, 273–295.

[6] Peña Ramirez J, Fey RHB, Aihara K, Nijmeijer H 2014 An improved model for the classical Huygens' experiment on synchronization of pendulum clocks. *J. Sound Vib.* 333, 7248–7266. (doi:10.1016/j.jsv.2014.08.030)

[7] Peña Ramirez J, Olvera LA, Nijmeijer H, Alvarez J 2016 The sympathy of two pendulum clocks: beyond Huygens' observations. *Sci. Rep.* 6, 23580 (doi:10.1038/srep23580) 27020903 10.1038/srep23580 PMC4810368

[8] Strogatz SH, Stewart I 1993 Coupled oscillators and biological synchronization. *Sci. Am.* 269, 102–109. (doi:10.1038/scientificamerican1293-102) 8266056 10.1038/scientificamerican1293-102

[9] Pikovsky A, Rosenblum M, Kurths J 2001 *Synchronization: a universal concept in nonlinear sciences*. Cambridge, UK: Cambridge University Press.

[10] Blekhman II 1988 *Synchronization in science and technology* (translated by E.I. Rivin) New York, NY: ASME Press.

[11] Pikovsky A, Maistrenko YL 2003 *Synchronization: theory and application*. NATO Science Series II, vol. 109 Dordrecht, The Netherlands: Springer.

[12] Strogatz S 2003 **Sync: the emerging science of spontaneous order**. New York, NY: Theia.

[13] Manevich AI, Manevich LI 2005 *The mechanics of nonlinear systems with internal resonances*. London, UK: Imperial College Press.

[14] Osipov GV, Kurths J, Zhou C 2007 *Synchronization in oscilliatory networks*. Berlin, Germany: Springer.

[15] Boccaletti S 2008 *The synchronized dynamics of complex systems*. Amsterdam, The Netherlands: Elsevier Science.

[16] Leonovo G, Nijmeijer H, Pogromsky A, Fradkov A (eds). 2010 Dynamics and control of hybrid mechanical systems. In *Proc. ENOC-2008, Saint Petersburg, Russia, 30 June–4 July 2008*. Singapore: World Scientific.

[17] Luo ACJ 2013 **Dynamical system synchronization**. New York, NY: Springer.

[18] Huygens C 1673 *Horologium Oscillatorium Sive de Motu Pendulorum ad Horologia Aptato Demonstrationes Geometricae*. Paris, France: Apud F. Muguet.

[19] Newton I 1687 *Philosophi Naturalis Principia Mathematica*. London, UK: Jussu Societatis Regi.

[20] Ellicott J 1739 An account of the influence which two pendulum clocks were observed to have upon each other. *Phil. Trans.* 41, 126–128. (doi:10.1098/rstl.1739.0014)

[21] Ellicott J 1739 Further observations and experiments concerning the two clocks above-mentioned. *Phil. Trans.* 41, 128–135. (doi:10.1098/rstl.1739.0015)

[22] Pantaleone J 2002 Synchronization of metronomes. *Am. J. Phys.* 70, 992–1000. (doi:10.1119/1.1501118)

[23] Senator M 2006 Synchronization of two coupled escapement-driven pendulum clocks. *J. Sound Vib.* 291, 566–603. (doi:10.1016/j.jsv.2005.06.018)

[24] Dilão R 2009 Antiphase and in-phase synchronization of nonlinear oscillators: the Huygens's clocks system. *Chaos* 19, 023118 (doi:10.1063/1.3139117) 19566253 10.1063/1.3139117

[25] Belykh VN, Pankratova EV, Pogromsky A, Nijmeijer H 2010 Two van der Pol-Duffing oscillators with Huygens coupling. In *Dynamics and control of hybrid mechanical systems*, *Proc. ENOC-2008, Saint Petersburg, Russia* (eds G Leonovo, H Nijmeijer, A Pogromsky, A Fradkov), pp. 181–194. Singapore: World Scientific.

[26] Belykh VN, Pankratova EV 2010 Chaotic dynamics of two van der Pol-Duffing oscillators with Huygens coupling. *Regul. Chaotic Dyn.* 15, 274–284. (doi:10.1134/S1560354710020140)

[27] Peña Ramirez J, Fey RHB, Nijmeijer H 2013 Synchronization of weakly nonlinear oscillators with Huygens' coupling. *Chaos* 23, 033118 (doi:10.1063/1.4816360) 24089954 10.1063/1.4816360

[28] Oliveira HM, Melo LV 2015 Huygens synchronization of two clocks. *Sci. Rep.* 5, 11548 (doi:10.1038/srep11548) 26204557 10.1038/srep11548 PMC4512151

[29] Golubitsky M, Stewart I, Schaeffer DG 1988 *Singularities and groups in bifurcation theory*, vol. II Berlin, Germany: Springer.

[30] Golubitsky M, Stewart I 2002 *The symmetry perspective*. Basel, Switzerland: Birkhauser.

[31] Kitanov P, Langford WF, Willms AR 2013 Double Hopf bifurcation with Huygens symmetry. *SIAM J. App. Dyn. Syst.* 12, 126–174. (doi:10.1137/110839461)

[32] Landes DS 1983 *Revolution in time: clocks and the making of the modern world*. Cambridge, UK: The Belknap Press of Harvard University Press.

[33] Golubitsky M, Schaeffer DG 1985 *Singularities and groups in bifurcation theory*, vol. I Berlin, Germany: Springer.

[34] Huygens C 1966 *Horologium oscillatorium: 1673*. London, UK: Dawsons.

[35] Czolczyński K, Perlikowski P, Stefański A, Kapitaniak T 2009 Clustering and synchronization of *n* Huygens' clocks. *Phys. A* 388, 5013–5023. (doi:10.1016/j.physa.2009.08.033)

[36] Golubitsky M, Stewart I 1985 Hopf bifurcation in the presence of symmetry. *Arch. Ration. Mech. Anal.* 87, 107–165. (doi:10.1007/BF00280698)

[37] Golubitsky M, Stewart I 2015 Recent advances in symmetric and network dynamics. *Chaos* 25, 097612 (doi:10.1063/1.4918595) 26428565 10.1063/1.4918595

[38] van Gils SA, Krupa M, Langford WF 1990 Hopf bifurcation with non-semisimple 1:1 resonance. *Nonlinearity* 3, 825–850. (doi:10.1088/0951-7715/3/3/013)

[39] Golubitsky M, Langford WF 1981 Classifications and unfoldings of degenerate Hopf bifurcations. *J. Diff. Eqn.* 41, 375–415. (doi:10.1016/0022-0396(81)90045-0)

[40] Guckenheimer J, Holmes P 1983 *Nonlinear oscillations, dynamical systems, and bifurcation of vector fields*. Berlin, Germany: Springer.

[41] Kitanov P 2011 Normal form analysis for bifurcations with Huygens symmetry. PhD thesis, University of Guelph, Guelph, Canada.

[42] Willms AR, Kitanov PM, Langford WF 2017 Data from: Huygens' clocks revisited Dryad Digital Repository. (doi:10.5061/dryad.p4sg5) 10.1098/rsos.170777 PMC5627120 28989780

[43] Willms AR 2013 Breathing torus near double Hopf bifurcation. See https://dsweb.siam.org/Media-Gallery/breathing-torus-near-double-hopf-bifurcation. DSWeb Magazine (www.dynamicalsystems.org), Media Gallery Entry.

[44] Iooss G, Langford WF 1980 Conjectures on the routes to turbulence via bifurcations. *Ann. NY Acad. Sci.* 357, 489–505. (doi:10.1111/j.1749-6632.1980.tb29712.x)

[45] Kuznetsov Y 2004 *Elements of applied bifurcation theory*. Berlin, Germany: Springer.

[46] Ruelle D, Takens F 1971 On the nature of turbulence. *Commun. Math. Phys.* 20, 167–192. (doi:10.1007/BF01646553)

[47] Jost R, Zehnder E 1972 A generalization of the Hopf bifurcation theorem. *Helv. Phys. Acta* 45, 258–276.

[48] Chenciner A, Iooss G 1979 Persistence et bifurcation de torus invariants. *Arch. Rat. Mech. Anal.* 71, 301–306. (doi:10.1007/BF00247705)

[49] Chow SN, Li C, Wang D 1994 *Normal forms and bifurcation of planar vector fields*. Cambridge, UK: Cambridge University Press.

[50] Feeman TG 2000 Equal area world maps: a case study. *SIAM Rev.* 42, 109–113. (doi:10.1137/S0036144599358997)

[51] Huygens C 1932 *Oeuvres complètes de Christiaan Huygens*, vol. 17 The Hague: Martinus Nijhoff Includes works from 1665.

[52] Huygens C 1986 *Christiaan Huygens' the pendulum clock or geometrical demonstrations concerning the motion of pendula as applied to clocks* (translated by R. Blackwell) Ames, IA: Iowa State University Press.

[53] Czolczyński K, Perlikowski P, Stefański A, Kapitaniak T 2011 Why two clocks synchronize: energy balance of the synchronized clocks. *Chaos* 21, 023129 (doi:10.1063/1.3602225) 21721771 10.1063/1.3602225

